# Recent Advances in the Search for Effective Anti-Alzheimer’s Drugs

**DOI:** 10.3390/ijms26010157

**Published:** 2024-12-27

**Authors:** Martyna Ogos, Dorota Stary, Marek Bajda

**Affiliations:** Department of Physicochemical Drug Analysis, Jagiellonian University Medical College, Medyczna Str. 9, 30-688 Kraków, Poland; martyna.ogos@student.uj.edu.pl (M.O.); dorota.stary@doctoral.uj.edu.pl (D.S.)

**Keywords:** Alzheimer’s disease, multi-target-directed ligands, β-amyloid aggregation inhibitors, AChE inhibitors, donepezil, tacrine, BChE inhibitors, neurodegenerative disorders

## Abstract

Alzheimer’s disease, the most common form of dementia, is characterized by the deposition of amyloid plaques and neurofibrillary tangles in the brain, leading to the loss of neurons and a decline in a person’s memory and cognitive function. As a multifactorial disease, Alzheimer’s involves multiple pathogenic mechanisms, making its treatment particularly challenging. Current drugs approved for the treatment of Alzheimer’s disease only alleviate symptoms but cannot stop the progression. Moreover, these drugs typically target a single pathogenic mechanism, leaving other contributing factors unaddressed. Recent advancements in drug design have led to the development of multi-target-directed ligands (MTDLs), which have gained popularity for their ability to simultaneously target multiple pathogenic mechanisms. This paper focuses on analyzing the activity, mechanism of action, and binding properties of the anti-Alzheimer’s MTDLs developed between 2020 and 2024.

## 1. Introduction

### 1.1. Pathogenesis of Alzheimer’s Disease

**Figure 1:**
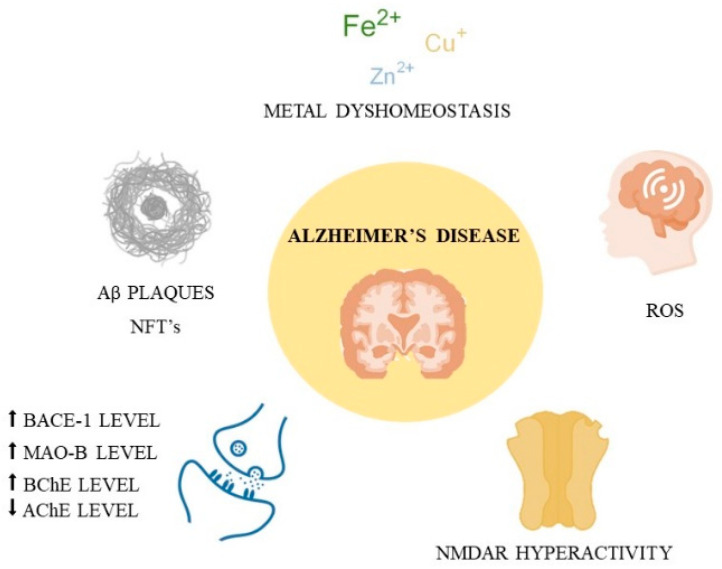
Pathogenesis of Alzheimer’s disease.

Dementia is one of the most common health-related problems worldwide, affecting more than 10 million people each year [1]. Alzheimer’s disease (AD), which accounts for up to 70% of cases of dementia, is a neurodegenerative disorder characterized by the degeneration of synapses and neuronal atrophy in the hippocampus and cerebral cortex, leading to cognitive decline and progressive memory impairment [2]. Both genetics and environmental factors play a crucial role in the occurrence of the disease. Unfortunately, the exact pathogenesis of this illness is still unknown [2]. However, several aspects of its pathogenesis have been identified, including deposition of beta-amyloid plaques and neurofibrillary tangles, dysfunction of cholinergic transmission, monoamine oxidase B (MAO-B) and beta-site amyloid precursor protein cleaving enzyme 1 (BACE1) overexpression, *N*-methyl-*D*-aspartate receptor (NMDAR) overactivity, reactive oxygen species (ROS) production, and dysregulation of metal homeostasis (Figure 1) [3].

Regarding beta-amyloid deposition, Aβ plaques are created by proteolytic cleavage of amyloid precursor protein (APP), mediated by β-secretase and γ-secretase [3]. Aβ_42_ is more hydrophobic and more prone to aggregate than Aβ_40_, making it the more neurotoxic form, and the major component of senile plaques [4]. While a healthy brain contains mostly Aβ_40_, in AD patients, the ratio of Aβ_42_/Aβ_40_ increases [4]. The polymerization of Aβ peptides into fibrils and plaques disrupts cholinergic synaptic signaling and activates the kinases, leading to hyperphosphorylation of the tau protein and the formation of neurofibrillary tangles (NFTs) [5]. The accumulation of these aggregates causes local inflammatory responses, resulting in neurotoxicity [5]. Aβ oligomers are also considered factors that can destroy the integrity of the cell membrane, leading to cell death [3]. Some authors suggest that Aβ can also agitate calcium channels, causing an increased level of calcium in the cells [4]. Calcium overload results in mitochondria failure, lipid peroxidation, and the generation of free radicals [4].

Another factor influencing AD progression is the dysfunction of cholinergic transmission [6]. The cholinergic system is essential for maintaining normal cognitive functions, and degeneration of central cholinergic neurons (observed in AD) triggers learning and memory dysfunction [6]. Acetylcholine (ACh) is an excitatory neurotransmitter of the cholinergic pathways, and its levels are reduced in patients with AD-induced dementia [7]. Acetylcholinesterase (AChE) and butyrylcholinesterase (BChE) are enzymes involved in the hydrolysis of ACh, preventing excess levels of the neurotransmitter in the synapses [7]. With the progression of Alzheimer’s disease, AChE levels decrease, and BChE levels increase, while in healthy brains, AChE is the predominant hydrolyzing enzyme [7]. AChE also contributes to Aβ aggregation by interacting with both the aggregates and with Aβ peptides, thereby increasing their neurotoxicity [8].

The role of monoamine oxidase (MAO) in the pathogenesis of AD is under investigation. MAO is an enzyme involved in the metabolism of monoamine neurotransmitters and amines [9]. The enzyme exists in two forms, MAO-A and MAO-B, and both are located in the brain [9]. The levels of MAO-B are enhanced in the hippocampus and cerebral cortex of patients with AD compared to healthy individuals [9]. The overexpression of MAO-B contributes to the development of oxidative stress by inducing the generation of free radicals and hydrogen peroxide [9]. Elevated levels of MAO-B also have an impact on amyloid plaque production [9].

Moving on to the *N*-methyl-*D*-aspartate receptors (NMDARs), these cationic channels are gated by glutamate [10] and play a key role in synaptic plasticity, which is impaired in the AD brain and is closely linked to cognitive decline [11]. Excessive NMDAR activity leads to excitotoxicity and prelude cell death and neurodegeneration [11].

Looking into the neuroinflammation present in AD, it is worth mentioning metal dyshomeostasis. Transition metals such as copper, iron, and zinc (whose levels are elevated in the AD brain) can ease the generation of free radicals and take part in inflammation [12]. The Fenton reaction between reduced transition metals and hydrogen peroxide yields the hydroxyl radical, a highly reactive oxygen species. Furthermore, transition metals can accelerate Aβ aggregation and contribute to its toxicity [12]. Iron binding to Aβ or tau induces the formation of beta-amyloid plaques and neurofibrillary tangles while also inducing their neurotoxicity [13]. Similarly, copper ions can induce the generation of Aβ aggregates. Senile plaques containing copper can interact with the lipid bilayer and thus increase the permeability of membranes [13]. Excessive zinc ion levels can also induce Aβ production [13]. In addition, elevated levels of zinc in mitochondria can increase reactive oxygen species production [13].

β-secretase, also known as beta-site amyloid precursor protein cleaving enzyme (BACE1), together with γ-secretase, is involved in the cleavage of amyloid precursor protein (APP) [14]. This process results in Aβ species formation and their further aggregation. BACE1 initiates the cleavage of APP, precisely at the Asp1 and Glu11 sites of Aβ, leading to the formation of membrane-bound C-terminal fragments called C99 and C89, respectively [14]. Elevated levels of β-secretase in the AD brain contribute to excess amyloid plaques [14].

### 1.2. Current Treatments for Alzheimer’s Disease

**Figure 2:**
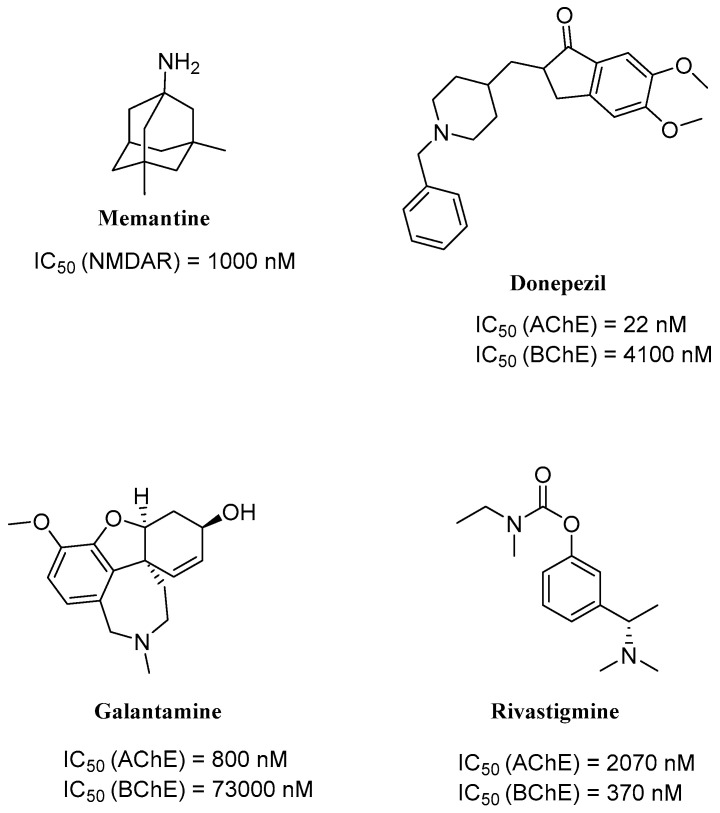
Structures and activities of approved anti-Alzheimer agents.

Currently, there are only a few drugs that are approved by the Food and Drug Administration for AD therapy [15]. On the market are three cholinesterase inhibitors and one NMDA antagonist. While these drugs can only reduce symptoms of the disease, such as memory impairment and cognitive decline, they do not stop the progression of AD [15]. Cholinesterase inhibitors increase cholinergic function via inhibition of enzymes responsible for the breakdown of acetylcholine (Ach) [16]. The approved drugs from this group are donepezil (IC_50_ for AChE is 22 nM and for BChE is 4100 nM [4]), galantamine (IC_50_ for AChE is 800 nM and for BChE is 73,000 nM [4]), and rivastigmine (IC_50_ for AChE is 2070 nM and for BChE is 370 nM [17]) (Figure 2). Donepezil and galantamine are reversible AChE inhibitors. Donepezil is highly selective for AChE [15]. On the other hand, rivastigmine is a pseudoreversible inhibitor of both AChE and BuChE [15]. However, as the disease progresses, these drugs become less effective due to the degeneration of Ach-producing neurons [18]. The group of NMDA antagonists includes memantine (IC_50_ is around 1000 nM [19]) (Figure 2). This drug can be combined with donepezil as an extended-release preparation for combination therapy [20]. Memantine is an antagonist of NMDARs and acts as an open-channel blocker, which prevents the overactivation and neurotoxicity induced by glutamate excess [21].

Apart from small-molecule drugs, there are two monoclonal antibodies that target amyloid-beta (Aβ) [22]. They belong to the group of medications whose aim is to modify the pathological steps causing AD, helping to postpone or stop its progression; therefore, this group of drugs is called “disease-modifying” [22]. The two human monoclonal antibodies mentioned above are Aducanumab (the first disease-modifying drug approved by the FDA in 2021) and Lecanemab (approved by the FDA in 2023) [23,24]. Aducanumab targets soluble *β*-amyloid oligomers and insoluble aggregates (fibrils and β-amyloid plaques) and works by removing beta-amyloid from the brain [24]. Lecanemab mainly targets the protofibrils of beta-amyloid. In phase 2b clinical trials, Lecanemab showed an impact on alleviating cognitive decline and had a low incidence of amyloid-related imaging abnormalities (ARIAs) [23]. Aducanumab in higher doses also had some impact on alleviating cognitive decline but had a much higher incidence of ARIAs [24].

Overall, the available medications for AD treatment cannot stop the progression of the disease; they can only help to manage the symptoms and slightly improve the quality of life of patients with AD.

### 1.3. Multi-Target-Directed Ligands in Alzheimer’s Disease

Available drugs for AD treatment can be classified as target-specific drugs (TSDs), which target only one of many pathological factors [25]. These kinds of medications are not especially successful in treating multifactorial diseases like AD [26]. In medicinal chemistry, a new trend called “polypharmacology” using multi-target-directed ligands (MTDLs) is considered more effective for diseases with multiple pathogenesis [26]. MTDLs are designed by incorporating at least two pharmacophores into one molecule [27]. They can be divided into three groups based on their structure: linked, when two active molecules are connected using a linker; fused, if they are directly attached to each other; or merged, in the case of overlapping molecules [28]. Although MTDLs are superior to TSDs, the same effect can be achieved using combination therapies containing several TSDs [27]. Nevertheless, MTDL therapy has more advantages than combination therapy [27]. First, the application of multifunctional compounds limits the risk of drug–drug interactions and the risk of adverse drug reactions. Additionally, the dosage recommendations are simpler [29,30,31].

**Figure 3:**
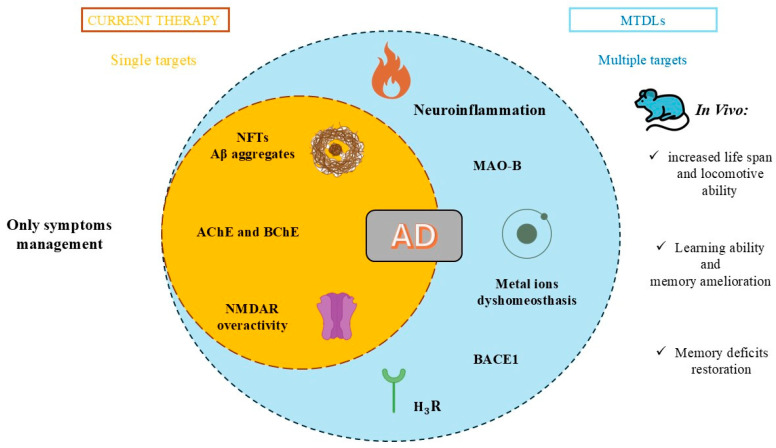
Comparison of current therapies for Alzheimer’s disease with MTDL approaches.

Currently, the most popular MTDL strategy for potential AD treatment involves the inhibition of ChE combined with the influence on other targets [32]. The most numerous compounds can inhibit AChE and/or BuChE and stop AChE-induced or metal-induced aggregation as well as self-aggregation of beta-amyloid [32]. The structural fragments of the MTDLs involved in AChE inhibition are mostly derived from donepezil or tacrine (Figure 3) [32]. As mentioned above, donepezil is a selective and reversible AChE inhibitor, while tacrine is a reversible inhibitor of both cholinesterases (ChEs) [4]. Unfortunately, due to its hepatotoxicity, tacrine is no longer available on the market for AD patients [33]. Combining tacrine with another structure followed by physicochemical optimization can lower or prevent the hepatotoxicity connected with tacrine fragments [32]. MTDLs that do not target ChE inhibition often influence NMDARs and MAOs or have chelation properties combined with senile plaque suppression [32].

In this review, we present the most recent progress in the field of MTDL approaches for AD treatment (Figure 3). We describe the most important multifunctional compounds published from 2020 to early 2024. Collected compounds are grouped based on their major mechanism of action, which includes cholinesterase inhibition, histamine receptor antagonism, beta-amyloid and tau aggregation inhibition, metal ion chelating, reactive oxygen species scavenging, monoamine oxidase inhibition, β-secretase inhibition, and *N*-methyl-*D*-aspartate antagonism (Table 1). In addition to their main activity, all of the compounds target other pathological factors present in Alzheimer’s disease.

**Table 1:** Targets and key structural fragments responsible for the activity of MTDLs.

MTDL Group	MTDL Subgroup	Fragment Responsible for Main Biological Activity	Main Target	Other Targets
Multifunctional cholinesterase inhibitors	Donepezil-based cholinesterase inhibitors	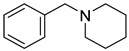 Benzylpiperidine (or its modification) [34,35,36,37,38,39,40,41,42,43,44,45,46,47]	AChE/BChE	Aβ aggregates [35,36,37,38,39,40,41,42,44,46], tau protein aggregates [38,39], AD-associated depression (SERT, 5-HT_1A_R) [34], metal dyshomeostasis (transition metals) [38,39,40,41,44,47], BACE1 [39], MAO-B [43], MAO-A [43], 5-HT_6_R [42], neuroinflammation (ROS [34,37,38,39,40,44,45,47], FAAH [36], UPS degradation pathway [41], HDAC [44], NRF2 [37,45], calcium channels [45])
	Tacrine-based cholinesterase inhibitors	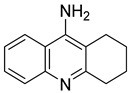 Tacrine (or its modification) [48,49,50,51,52,53,54] (Figure 4)		Aβ aggregates [50,52,53], tau protein aggregates [49,52], GSK-3β [49], DYRK1α [49], MAO-B [51,53], BACE1 [53,54], neuroinflammation (1L-1β [48], COX-2 [48], iNOS [48], ROS [49], TNF-κB [48]), metal dyshomeostasis (Fe^2+^, Cu^2+^, Zn^2+^) [50,53]
	Carbamate-based cholinesterase inhibitors	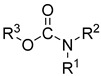 Carbamate [55,56]		Aβ aggregates [55,56], tau protein aggregates [56], MAO-B [56], APP [56], neuroinflammation (ROS [55], IL-6 [55], TNFα [55], iNOS [55], IL-4 [55])
	Cholinesterase inhibitors with diverse scaffolds	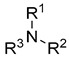 Tertiary amine [57] 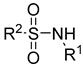 Sulfonamide [58] 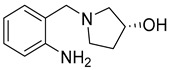 1-(2-aminobenzyl)pyrrolidin-3-ol [59] 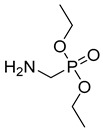 α-aminophosphonate [60] 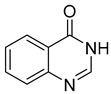 Quinazolin-4-one [60] 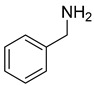 Benzylamine [61] 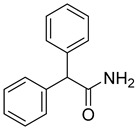 2,2-diphenylacetamide [61]		Aβ aggregates [57,59,61], metal dyshomeostasis (Cu^2+^) [57], BACE1 [61], GATs [61], neuroinflammation (DNA oxidation [60], calcium channel [58], NRF2 [58], TNFα [57], COX2 [57], iNOS [57], NO [57], IL-1β [57], ROS [58,59,60])
Multifunctional H_3_ receptor inverse agonists and antagonists		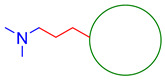 Blue—tertiary amine; Red—linear aliphatic ether linker; Green—aromatic, lipophilic, and bulky scaffold [62,63,64,65]	H_3_ receptor	AChE [62,63,64], BChE [62,63,64], MAO-B [62,63,64], Aβ aggregates [65], metal dyshomeostasis (Fe^2+^ and Cu^2+^) [65], neuroinflammation (ROS) [65]
Multifunctional tau and/or beta-amyloid aggregation inhibitors		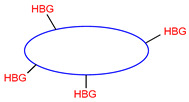 HBG—functional groups able to form hydrogen bonds; Blue—flat, aromatic, bulky, and hydrophobic core [66,67,68,69,70,71,72]	Aβ and tau aggregates	AChE [66,67,68], BChE [66], MAO-B [68], α-synuclein aggregates [70], GSK3β [71], DYRK1A [71], QC [72]
Multifunctional MAO-B inhibitors		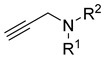 N-propargyl amine [73] 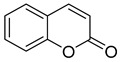 Coumarin [74]	MAO-B	Aβ aggregates [73], metal dyshomeostasis (Fe^3+^) [74], neuroinflammation (ROS) [74]
Multifunctional metal chelators and reactive oxygen species scavengers		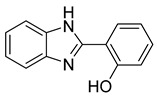 Hydroxyphenylbenzimidazole [75] 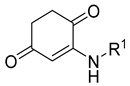 Substituted 2-aminocyclohex-2-ene-1,4-dione [76] 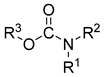 Carbamate [77] 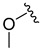 Methoxy group [77]	Metal ions and ROS	MAO-A [75], MAO-B [75], Aβ aggregation [75,76,77], neuroinflammation (IL-6 and TNFα) [76]
Multifunctional BACE1 inhibitors		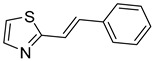 2-styrylthiazole [78] 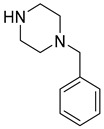 Benzylpiperazine [79] 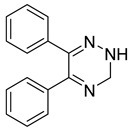 5,6-diphenyl-2,3-dihydro-1,2,4-triazine [80]	BACE1	AChE [78,79,80], BChE [79], Aβ aggregates [78,79,80], DYRK1A [80]
Multifunctional NMDAR blockers		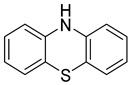 Phenothiazine [81] 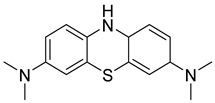 Methylene blue derivative [81,82] 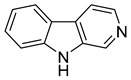 β-carboline (or its modification) [82]	NMDAR	AChE [81,82], BChE [81], tau protein aggregates [82]

## 2. Multifunctional Cholinesterase Inhibitors

**Figure 4:**
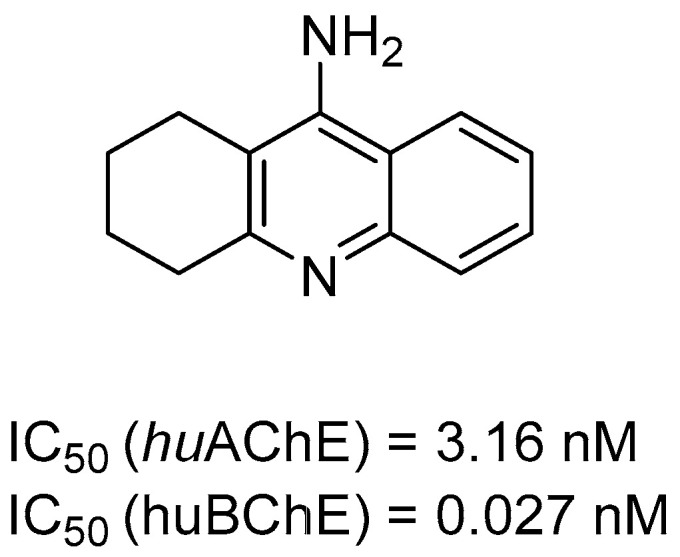
Structure of tacrine.

**Figure 5:**
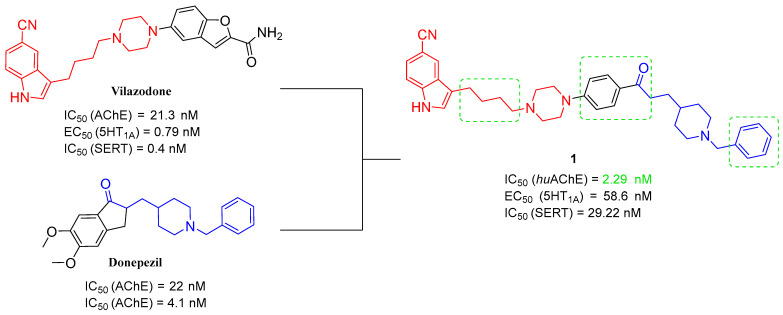
Compound **1** derived from vilazodone and donepezil. Green shapes indicate fragments that contribute to the highest huAChE inhibition (four-methylene linker, unsubstituted phenyl ring, and *para*-substituted benzoyl).

**Figure 6:**
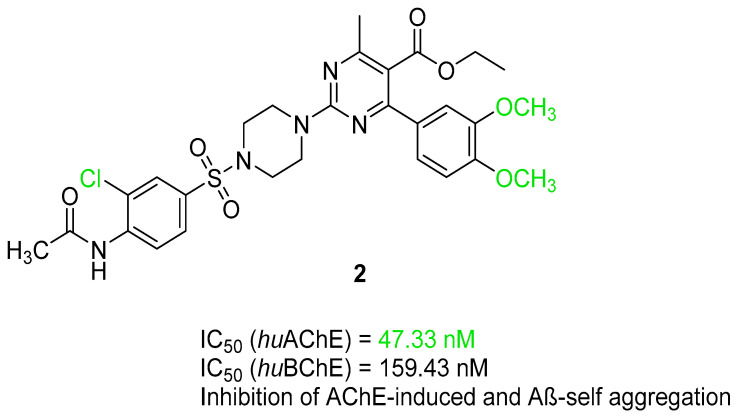
Structure of compound **2**. The green color indicates fragments that contribute to the highest *hu*AChE inhibition (two methoxy groups and chlorine atom).

**Figure 7:**
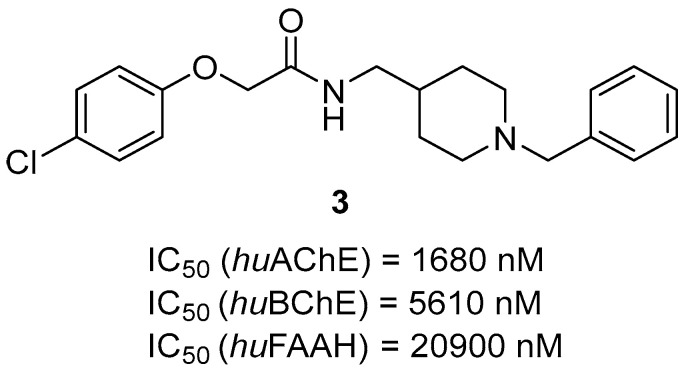
Structure of compound **3**.

**Figure 8:**
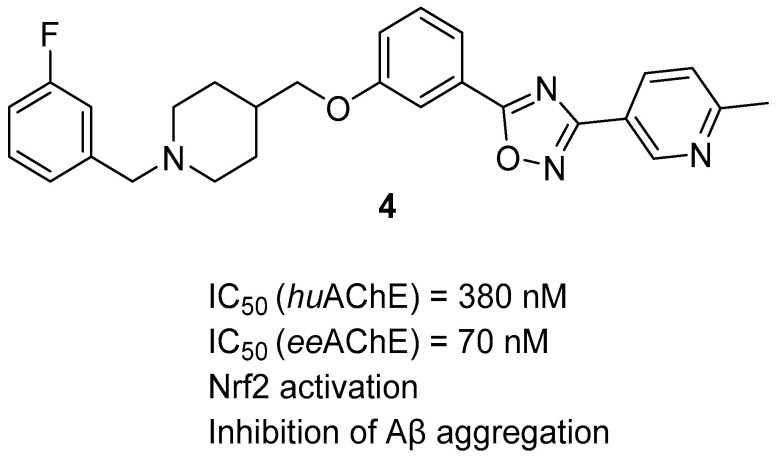
Structure of compound **4**.

**Figure 9:**
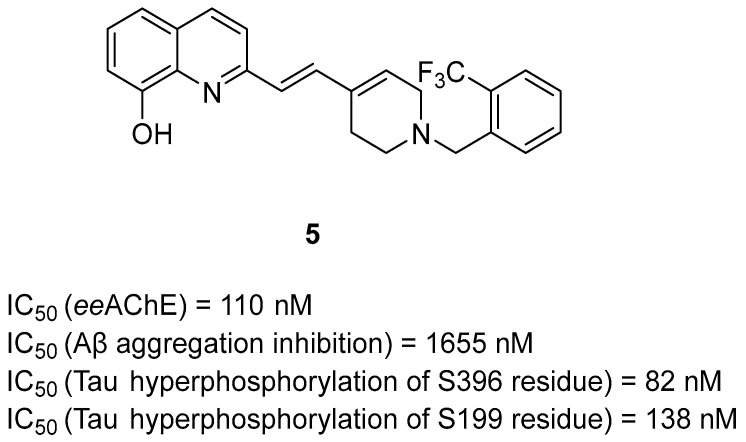
Structure of compound **5**.

**Figure 10:**
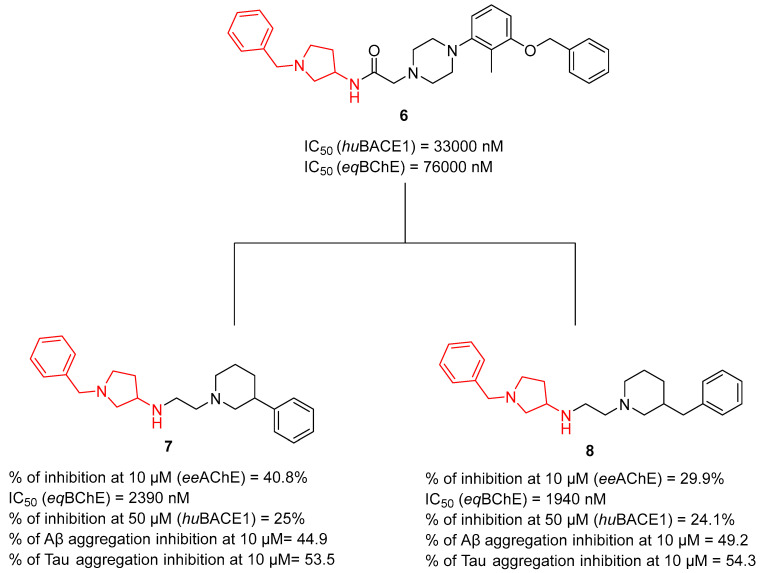
Structures of compounds **6**, **7**, and **8**.

**Figure 11:**
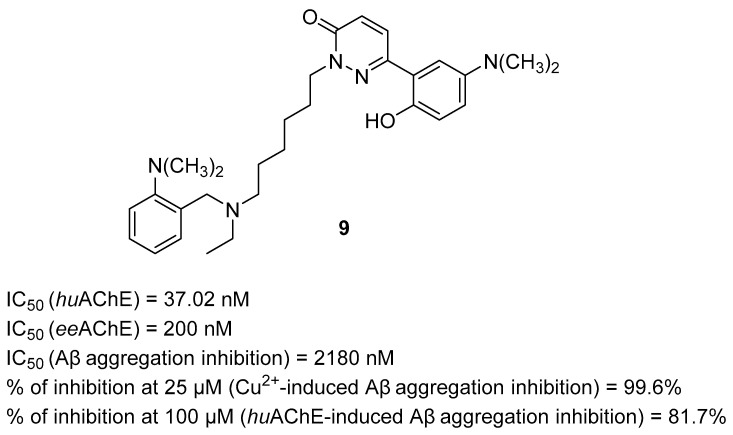
Structure of compound **9**.

**Figure 12:**
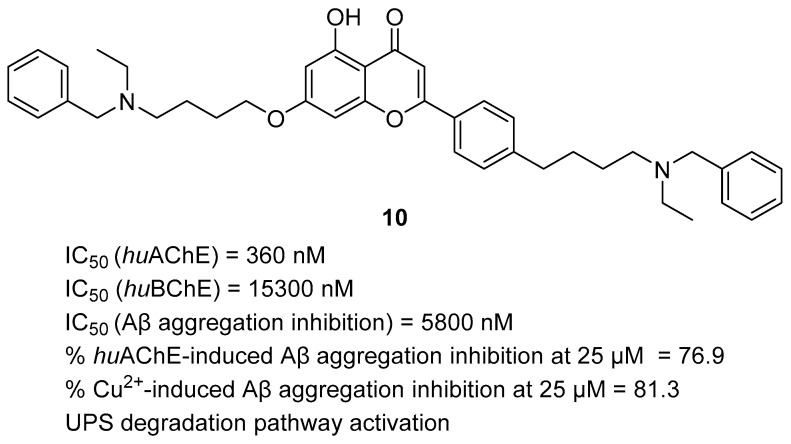
Structure of compound **10**.

**Figure 13:**
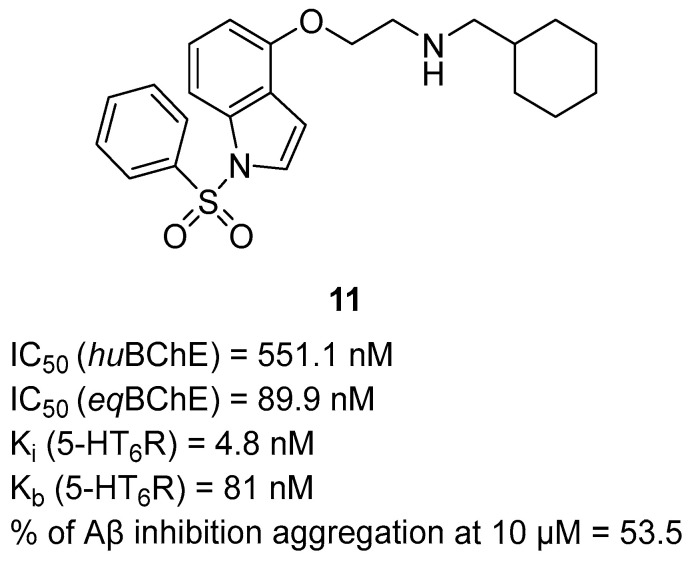
Structure of compound **11**.

**Figure 14:**
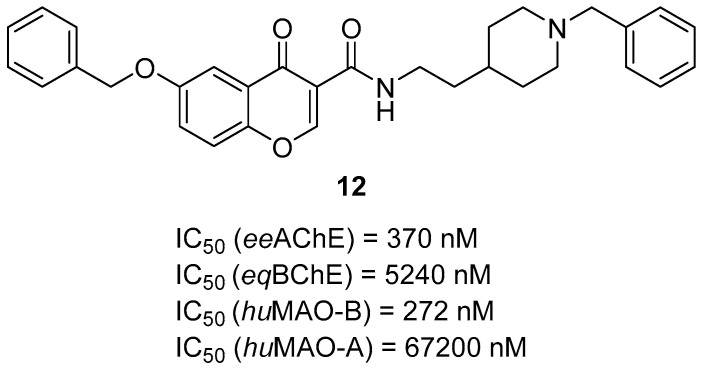
Structure of compound **12**.

**Figure 15:**
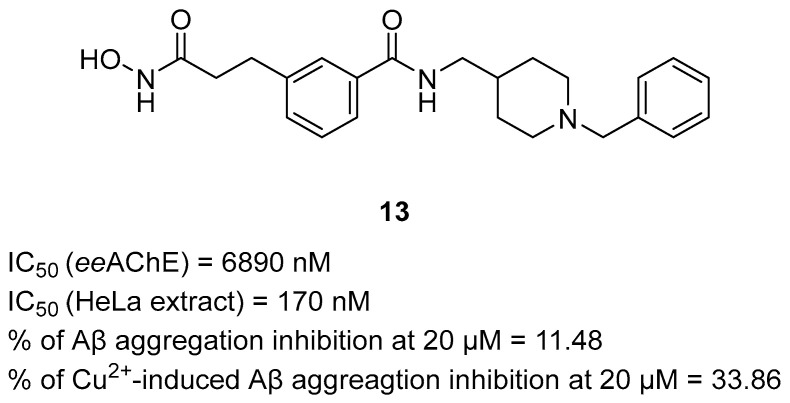
Structure of compound **13**.

**Figure 16:**
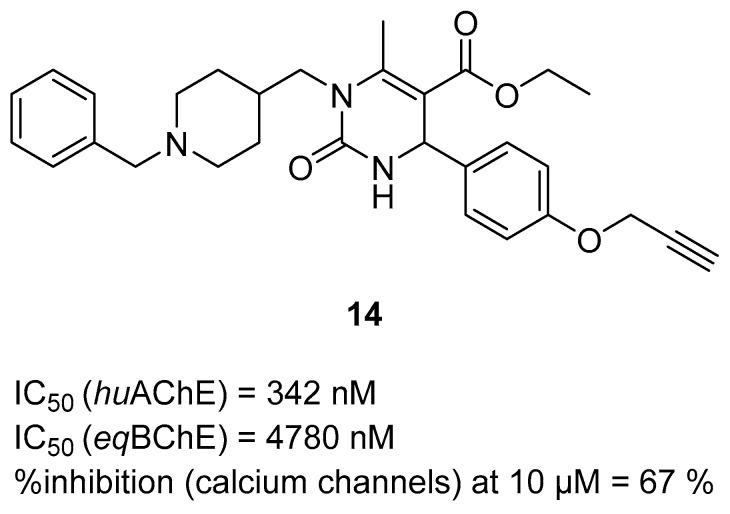
Structure of compound **14**.

**Figure 17:**
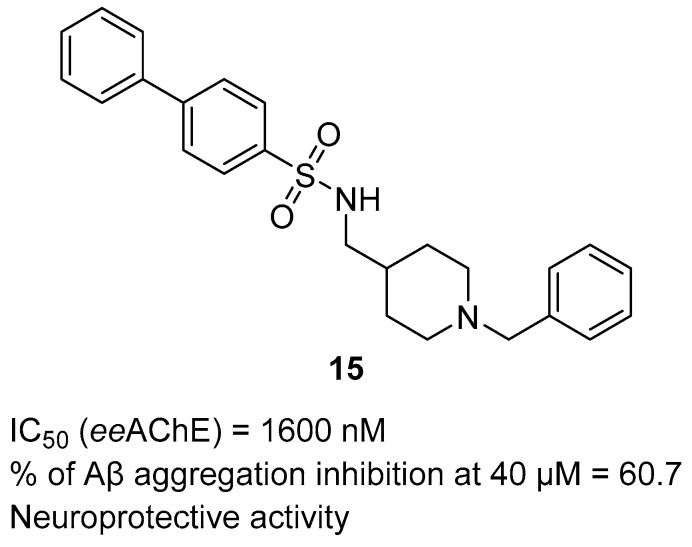
Structure of compound **15**.

**Figure 18:**
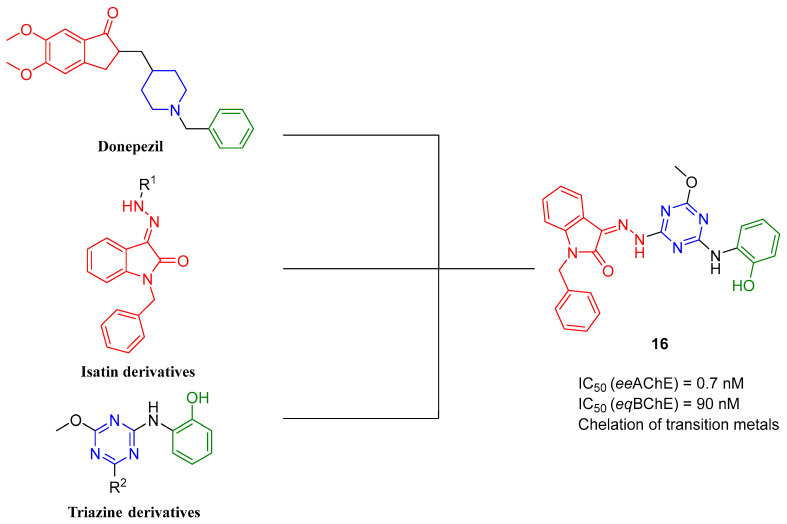
Compound **16** derived from isatin derivatives, triazine derivatives, and donepezil. Colors indicate similar structural motifs.

**Figure 19:**
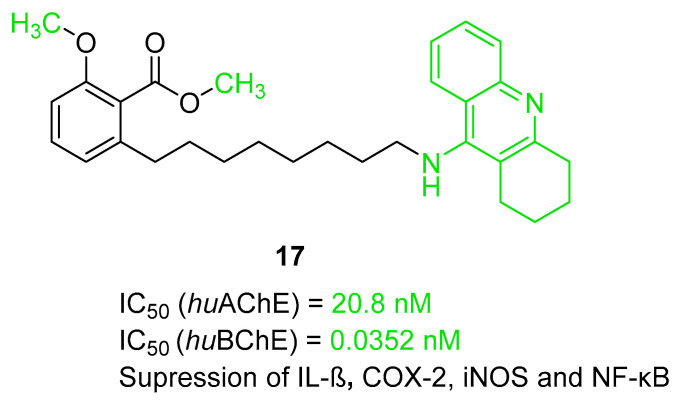
Structure of compound **17**. The green color indicates fragments that contribute to the highest *hu*ChE inhibition (methyl substituents at carboxylic and phenolic groups and tacrine).

**Figure 20:**
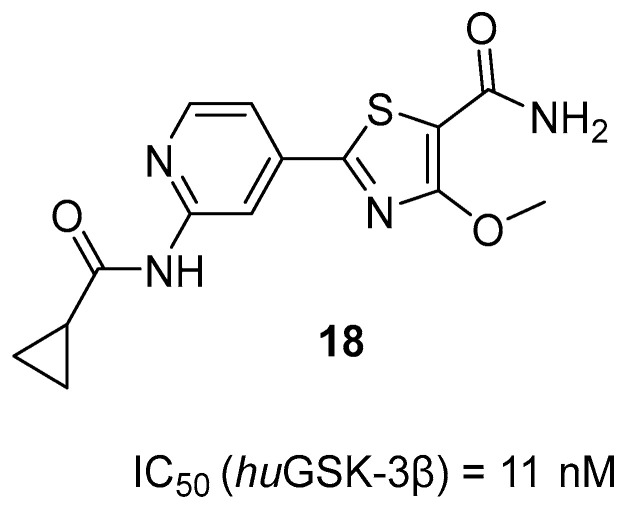
Structure of compound **18**.

**Figure 21:**
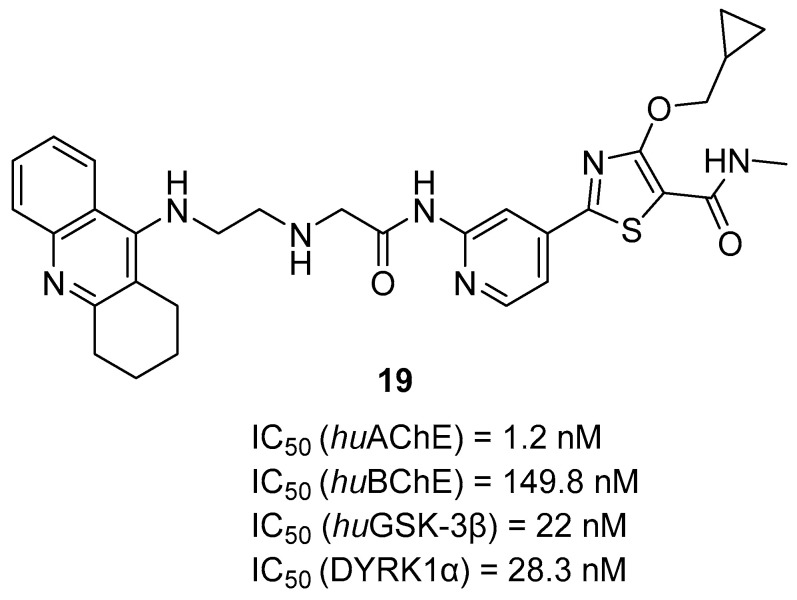
Structures of compound **19**.

**Figure 22:**
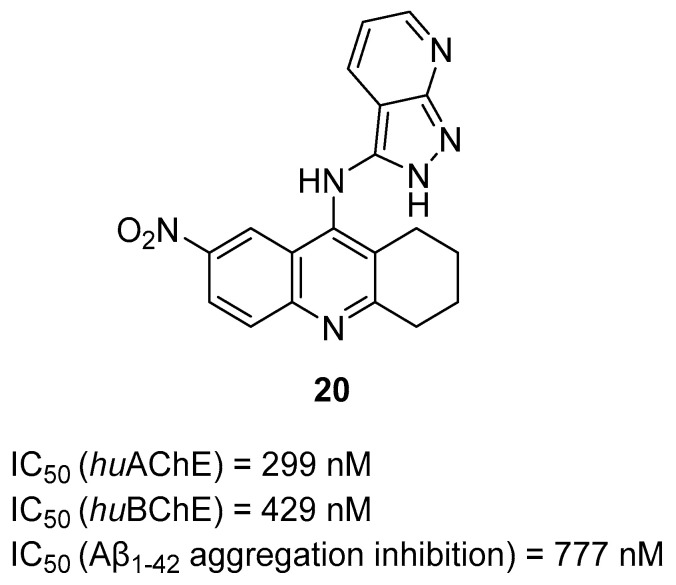
Structure of compound **20**.

**Figure 23:**
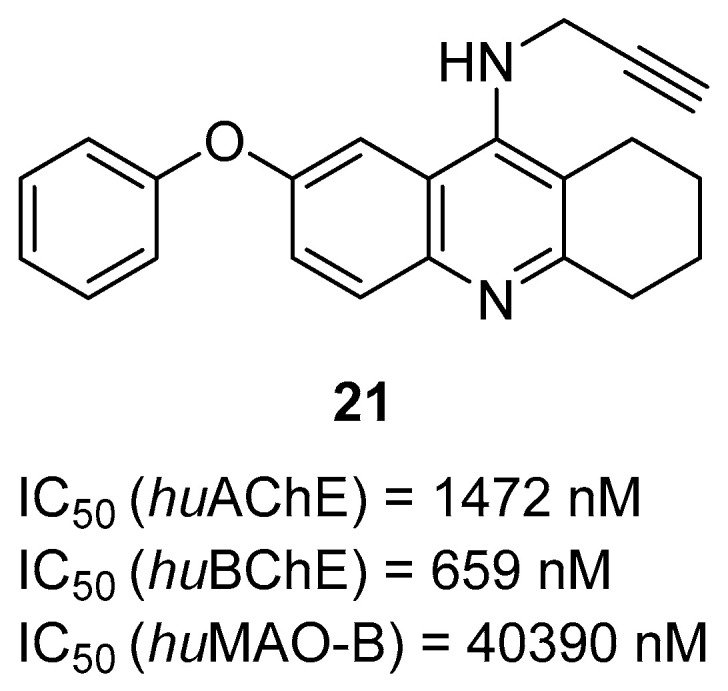
Structure of compound **21**.

**Figure 24:**
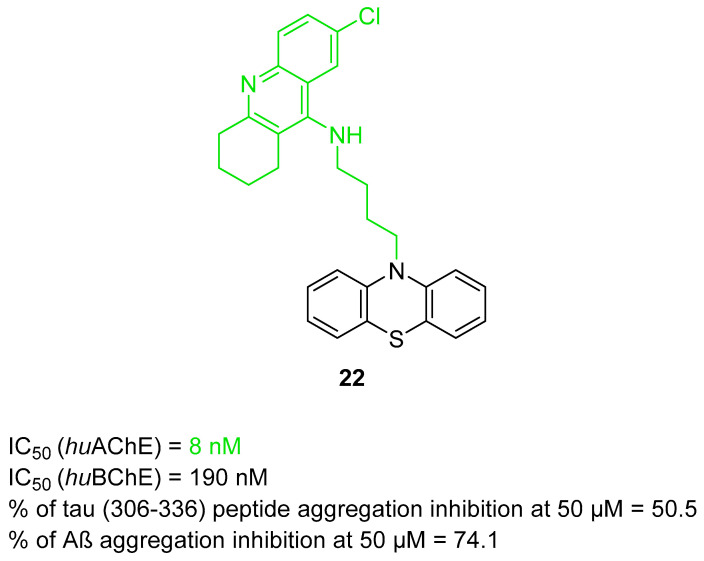
Structure of compound **22**. The green color indicates fragments that contribute to the highest *hu*AChE inhibition (four-methylene linker and 6-chlorotacrine).

**Figure 25:**
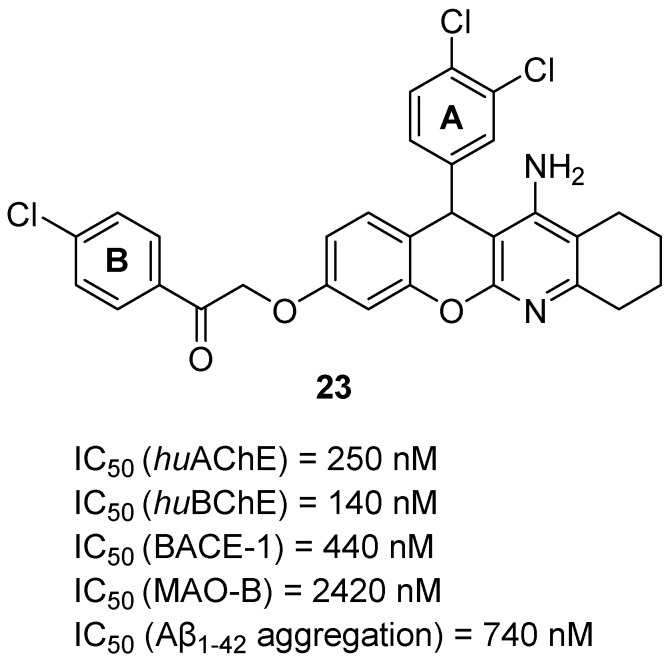
Structure of compound **23**.

**Figure 26:**
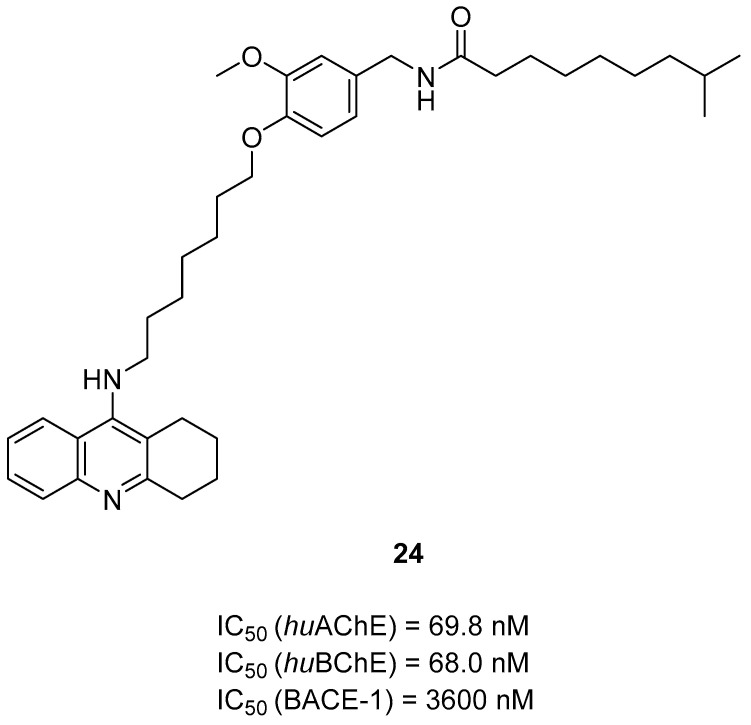
Structure of compound **24**.

**Figure 27:**
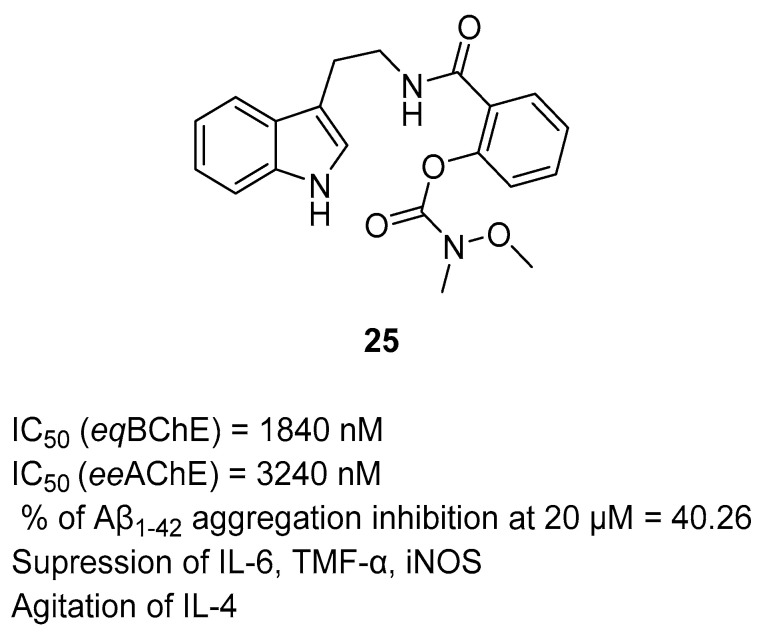
Structure of compound **25**.

**Figure 28:**
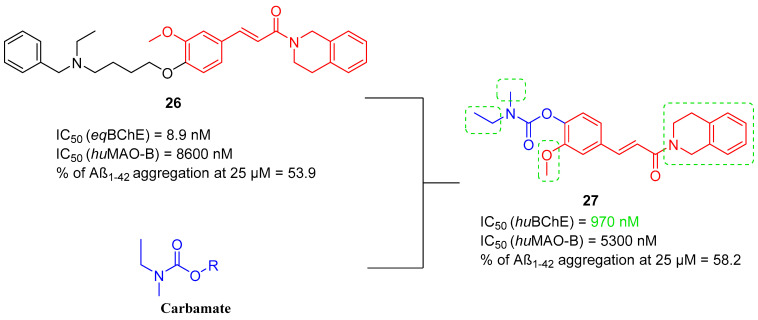
Design of compound **27**. Green shapes indicate fragments that contribute to the highest *hu*BChE inhibition (alkyl substituents at carbamate, methoxy substituent of ferulic acid, and 1,2,3,4-tetrahydroisoquinoline).

**Figure 29:**
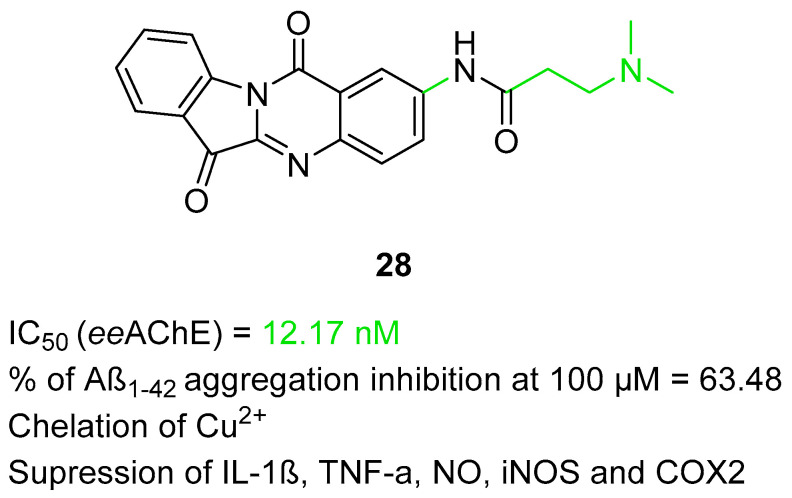
Structure of compound **28**. The green color indicates fragments that contribute to the highest *ee*AChE inhibition (substitution of tryptanthrin at position 2, ethylene linker, and amine group substituted with two methyl groups).

**Figure 30:**
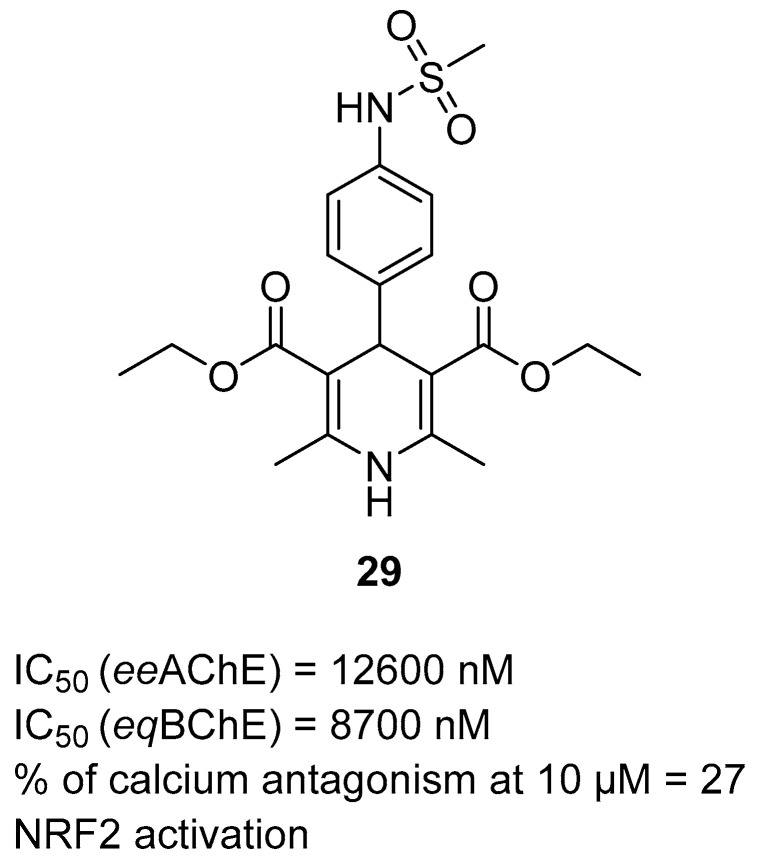
Structure of compound **29**.

**Figure 31:**
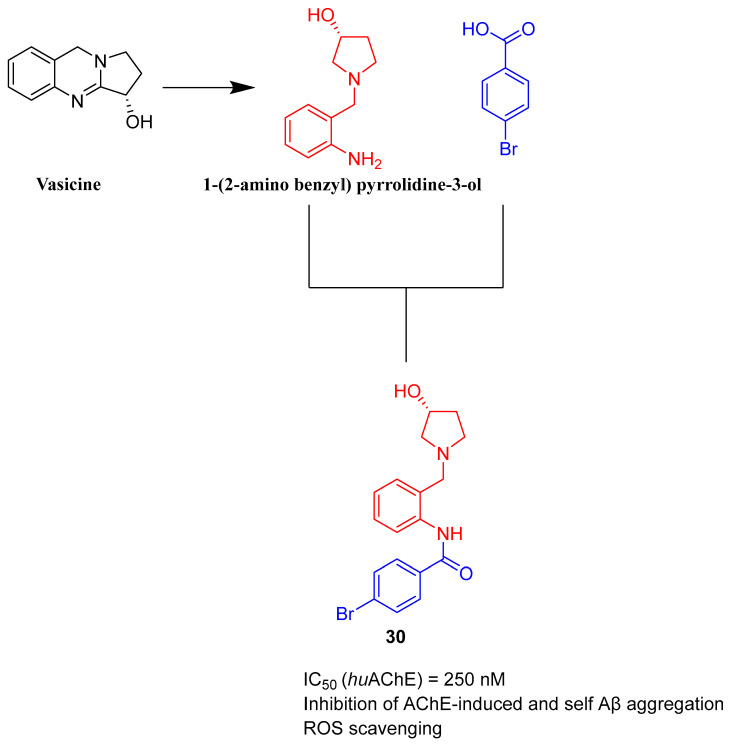
Design of compound **30** based on 1-(2-aminobenzyl)pyrrolidine-3-ol, derived from vasicine.

**Figure 32:**
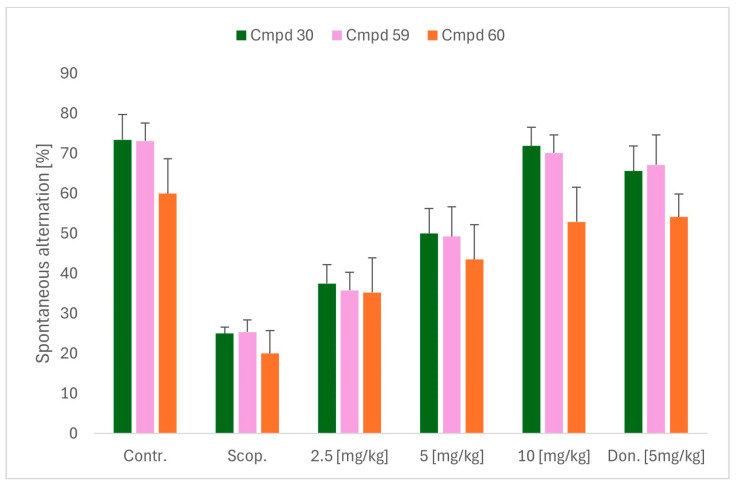
Effects of compounds **30**, **59**, and **60** (at doses of 2.5, 5, and 10 mg/kg) on scopolamine-administrated (Scop.) cognitive dysfunction mice, with donepezil (Don.) as reference in 5 mg/kg. Data are shown as mean ± SD. Each compound has its own positive and negative control as well as reference.

**Figure 33:**
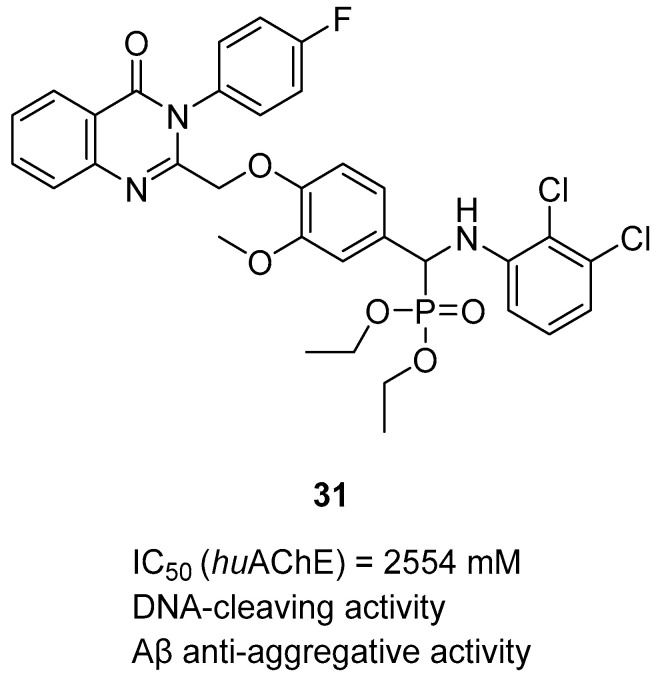
Structure of compound **31**.

**Figure 34:**
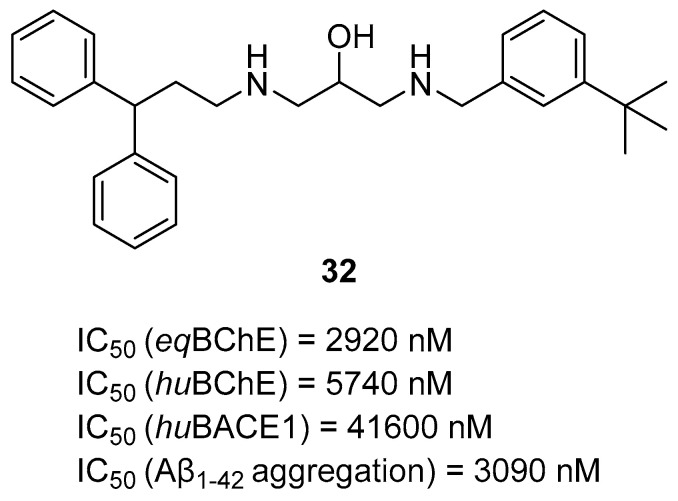
Structure of compound **32**.

**Figure 35:**
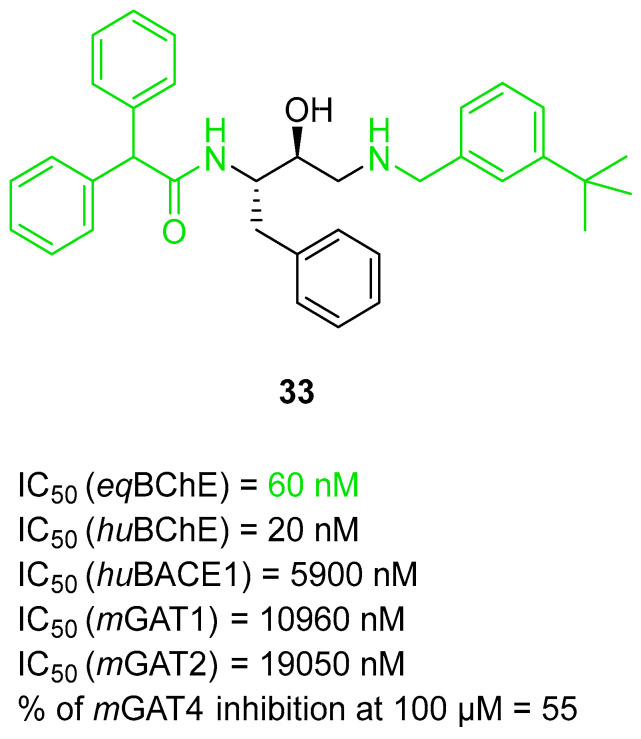
Structure of compound **33**. The green color indicates fragments that contribute to the highest *eq*BChE inhibition (direct connection of benzhydryl moiety with amide group, amide group itself, and bulky substituent at benzylamine).

As mentioned, cholinergic neurotransmission is crucial for proper learning and memory functioning [6]. In this section, we collected multifunctional compounds whose main targets are acetylcholinesterase and/or butyrylcholinesterase. Compounds **1**–**16** (Figure 5, Figure 6, Figure 7, Figure 8, Figure 9, Figure 10, Figure 11, Figure 12, Figure 13, Figure 14, Figure 15, Figure 16, Figure 17 and Figure 18) [34,35,36,37,38,39,40,41,42,43,44,45,46,47] are based on donepezil, which is a selective human acetylcholinesterase (*hu*AChE) inhibitor with an IC_50_ value equal to 22 nM [4]). Two compounds from this group demonstrate significant human acetylcholinesterase and butyrylcholinesterase (*hu*ChE) inhibitory potency: molecule **1** (Figure 5), with an IC_50_ value for *hu*AChE equal to 2.29 nM [34] and molecule **2** (Figure 6), with IC_50_ values of 47.33 nM and 159.43 nM for *hu*AChE and *hu*BChE, respectively [35]. Molecules **17**–**24** (Figure 19, Figure 20, Figure 21, Figure 22, Figure 23, Figure 24, Figure 25 and Figure 26) [48,49,50,51,52,53,54] are derived from the structure of tacrine (Figure 4)—a non-selective inhibitor of cholinesterases (*hu*AChE with an IC_50_ of 3.16 nM and *hu*BChE with an IC_50_ of 0.027 nM [83]). Among tacrine derivatives, two MTDLs stand out: compound **17** (Figure 19), with an IC_50_ for *hu*AChE of 20.8 nM and an IC_50_ for *hu*BChE of 0.0352 nM [48], and compound **22** (Figure 24), with an IC_50_ value for *hu*AChE equal to 8 nM [52]. In turn, compounds **25** (Figure 27) [55] and **27** (Figure 28) [56] can suppress ChE via their carbamate fragments. The last derivatives, **28**–**33** (Figure 29, Figure 30, Figure 31, Figure 32, Figure 33, Figure 34 and Figure 35), are ChE inhibitors with diverse structural motifs [57,58,59,60,61]. Both carbamate-based and structurally diverse multifunctional cholinesterase inhibitors possess significantly lower potency towards *hu*ChE compared with derivatives of donepezil and tacrine.

### 2.1. Donepezil-Based Multifunctional Cholinesterase Inhibitors

Li et al. obtained a series of MTDLs by fusing (Figure 5) donepezil pharmacophore with a main moiety (substituted 1H-indole-5-carbonitrile) of vilazodone [34]. Vilazodone is an antidepressant, dual 5-HT_1A_ partial agonist, and serotonin transporter (SERT) inhibitor. The obtained chimeras act as AChE inhibitors, 5-HT_1A_ partial agonists, and SERT inhibitors. These drugs were designed to alleviate depression symptoms and ameliorate cognitive decline in AD patients at the same time. The most potent derivative is compound **1** (Figure 5), with stronger *hu*AChE inhibitory activity (IC_50_ = 2.29 nM) than donepezil (IC_50_ = 22 nM [4]). Conversion of the benzocyclopentanone (indanone) into a ketone linker improved both the 5-HT_1A_ agonism and SERT inhibition of compound **1** (EC_50_ reaching 58.6 nM for 5-HT_1A_ and IC_50_ = 29.22 for SERT). The lack of substituents at the phenyl ring of the donepezil fragment, four-methylene linker, the *para* substitution of carbonylbenzene, and the carbonylbenzene core itself improve the potency of hybrid 1 to all three targets. Subsequent assays indicated that derivative 1 is a mixed-type *hu*AChE inhibitor and displays fragile hERG inhibitory activity. Compound **1**, in comparison to donepezil, has much weaker inhibitory potency towards hERG (IC_50_ (compound **1**) > 40 µM and IC_50_ (donepezil) = 0.64 µM) and hence can be considered relatively safe from cardiotoxicity. Finally, the described compound can induce improvement in cognition and shows antidepressant potential.

Manzoor et al. developed phenylsulfonyl-pyrimidine derivatives. The skeleton was created by linking phenylpyrimidine and phenylsulfonyl fragments with the piperazine linker [35]. In the obtained compounds, the benzylpiperidine fragment of donepezil was modified. The piperidine was replaced with piperazine, and the methyl linker was replaced with the sulfonyl group. These compounds possess dual AChE and BChE inhibitory activity and, at the same time, can suppress Aβ aggregation. Compound **2** (Figure 6) is the most potent *hu*AChE inhibitor (IC_50_ = 47.33 nM) among all the synthesized molecules, exhibiting non-competitive inhibition. Its high potency is due to the presence of two electron-donating methoxy groups combined with one electron-withdrawing chlorine atom. This MTDL also strongly inhibits *hu*BChE (IC_50_ = 159.43 nM). Additionally, the described derivative can prevent both AChE-induced and self-aggregation of Aβ. Furthermore, MTDL **2** has neuroprotective effects and can mitigate oxidative stress.

Another MTDL series based on a donepezil fragment was synthesized by Brunetti et al. [36]. They obtained these hybrids by linking an aryloxyacetic fragment and benzylpiperidine moiety (of donepezil) with an amide linker. The hybrids act as BChE, AChE, and Fatty Acid Amide Hydrolase (FAAH) inhibitors. FAAH is an enzyme that hydrolyzes anandamide; its levels are elevated in astrocytes and microglia associated with neuritic plaques. Anandamide is an endocannabinoid, which, through activation of cannabinoid receptors, exerts neuroprotective and anti-inflammatory properties [84]. The increased levels of FAAH lead to higher levels of arachidonic acid, which are affiliated with prostaglandin synthesis and inflammation. The most potent and versatile of all targets is derivative **3** (Figure 7); hence, this compound was chosen as a representative [36]. It is a moderate *hu*AChE (IC_50_ = 1680 nM) and *hu*BChE (IC_50_ = 5610 nM) inhibitor. This level of inhibitory potency towards both ChEs can be associated with the absence of an additional electron-withdrawing group on the phenoxy ring (compounds bearing nitro groups exert much higher potencies). Compound **3** can also inhibit *hu*FAAH, with an IC_50_ of 20,900 nM. This weak potency can be correlated with a short methylene linker (derivatives bearing longer linkers exert higher *hu*FAAH inhibitory activity). Finally, compound **3** is capable of inhibiting the Aβ aggregation process slightly.

In 2022, Wang et al. obtained a new series of MTDLs by fusing the 1,2,4-oxadiazole core from the nuclear factor erythroid 2-related factor (Nrf2) activator with the benzylpiperidine fragment from donepezil [37]. These derivatives act as AChE inhibitors and Nrf2 activators. Activating the Nrf2 can alleviate inflammation and oxidative stress by acting on the Nrf2-ARE pathway. The most promising candidate is compound **4** (Figure 8), which is a strong, non-competitive electric eel acetylcholinesterase (*ee*AChE) inhibitor (IC_50_ = 70 nM) and a moderate *hu*AChE inhibitor (IC_50_ = 380 nM). Its potency towards *ee*AChE can be explained by the presence of a 1-benzyl-piperidine-4-yl fragment, substituted with a small electron-withdrawing group (fluorine atom) at position 3. Moreover, compound **4** is capable of increasing the level of Nrf2 and its downstream proteins (Heme Oxygenase-1, NAD(P)H Quinone Dehydrogenase 1, and Glutamate-Cysteine Ligase Modifier Subunit) in a dose-dependent manner by upregulating the expression of the mentioned factors. Thanks to this, derivative **4** can prevent neuroinflammation. In addition, the described MTDL possesses a protective effect against Aβ and H_2_O_2_ cytotoxicity and can inhibit ROS overproduction. Finally, compound **4** can ameliorate cognitive impairment.

Li et al. obtained a series of hydroxyquinoline-based compounds by linking benzyl-1,2,3,6-tetrahydropyridine (based on the benzylpiperidine fragment of donepezil) with the skeleton of hydroxyquinoline via a vinylene linker [38]. These MTDLs act as both AChE inhibitors and metal ion chelators. The most potent derivative is compound **5** (Figure 9), which is a strong, mixed-type *ee*AChE inhibitor with an IC_50_ of 110 nM. Overall, substitution on the benzene ring with electron-withdrawing groups (eventually slightly electron-donating) is highly beneficial for increased inhibitory activity towards *ee*AChE. On the other hand, the hydroxyquinoline moiety in derivative **5** is involved in metal chelation (Cu^2+^, Fe^3+^, Fe^2+^, Zn^2+^, and Al^3+^ ions). Furthermore, derivative **5** can inhibit *ee*AChE-induced and Cu^2+^-induced Aβ aggregation, and pretreatment with compound **5** can protect cells from mitochondrial damage and oxidative stress. The described compound can also reverse or prevent the phosphorylation of tau protein, ameliorate Aβ-induced cognitive impairment, and improve learning.

Another series of MTDLs in AD treatment was obtained by Wichur et al. They described a series of 1-benzpyrrolidyne-3-amine-based compounds [39]. This series was synthesized by optimizing compound **6** (Figure 10), which is a *hu*BACE1 (IC_50_ = 33,000 nM) and horse serum butyrylcholinesterase (*eq*BChE) (IC_50_ = 76,000 nM) inhibitor. Researchers kept the donepezil-based 1-benzylpyrrolidine-3-amine moiety of compound **6** unaltered and made changes, such as replacing the 3-(benzyloxy)-2-methylphenyl with benzyl and phenyl moieties, introducing substituted piperidine and reducing the amide bond. The obtained MTDLs act as BACE1, AChE, and BChE blockers; Aβ and tau aggregation inhibitors; and metal ion chelators. The most promising compounds were **7** and **8** (Figure 10). Both of these compounds are more active towards *eq*BChE (IC_50_ = 2390 nM for **7**, and 1940 nM for **8**) than *ee*AChE (inhibition of 40.8% for compound **7** and 29.9% for compound **8** at 10 µM). Derivative **8**, due to a benzyl substituent at the piperidine ring, was more potent towards *eq*BChE than compound **7**. Both of the compounds inhibit *hu*BACE1 by about 25% at 50 µM due to containing in their structure an amine linker instead of an amide linker and have the ability to inhibit Aβ and tau aggregation (around 50% at 10 µM for both compounds). Thanks to the presence of phenyl and benzyl substituents at the piperidine ring (at position 3) and an ethyleneamine linker (between piperidine and pyrrolidine) in the structure of the compounds, both of them can selectively chelate copper ions. Moreover, both compounds revealed significant antioxidant activity.

In 2022, Shi et al. obtained a series of 2-aminoalkyl-6-(2-hydroxyphenyl)pyridazin-3(2*H*)-one derivatives by linking the open-ring benzylpiperidine-based moiety with 6-(2-hydroxyphenyl)pyridazin-3(2H)-one by an alkane linker [40]. The obtained MTDLs act as AChE inhibitors, Aβ aggregation inhibitors, and metal ion chelators. The most potent compound from all series is **9** (Figure 11). This derivative is a selective, mixed-type *ee*AChE inhibitor, with an IC_50_ value equal to 200 nM, and a strong *hu*AChE inhibitor (IC_50_ = 37.02 nM). In addition, the electron-donating tertiary amine substituent at the *N*-benzylethylamine moiety and the same substituent at the hydroxyphenyl improve the potency of the compound towards *ee*AChE. The presence of dimethylamine substituent increases the electron density of phenolic hydroxyl moiety, leading to radical scavenging activity. Compound **9** can inhibit the production of reactive oxygen species induced by Cu^2+^. Further, dimethylamine attached to the hydroxyphenyl moiety is responsible for its outstanding ability to inhibit Cu^2+^-induced (percentage of inhibition is 99.6% at 25 µM), *hu*AChE-induced (percentage of inhibition is 81.7% at 100 µM), and self-aggregation of Aβ (IC_50_ = 2180 nM). The dimethylamine forms contacts with amyloid beta, disrupting peptide-peptide interactions and ensuring an anti-aggregatory effect. Furthermore, the presented MTDL promotes disaggregation of Cu^2+^-induced Aβ fibrils. In addition, derivative **9** is capable of chelating transition metal ions such as Cu^2+^, Fe^2+^, Fe^3+^, Zn^2+^, and Al^3+^. Finally, compound **9** possesses the ability to ameliorate memory decline by increasing acetylcholine levels in the cerebral cortex and by regulating oxidative stress.

Different donepezil-based MTDLs were created by Sang et al. They obtained a series of apigenin-based derivatives, which were made by linking apigenin or its analogs, such as naringenin, genistein, and chalcone, with modified benzylpiperidine (donepezil fragment) via a four-methylene linker [41]. Apigenin is a flavonoid with documented neuroprotective, antioxidant, and anti-inflammatory effects; also, it can modulate the ubiquitin–proteasome system. The ubiquitin–proteasome system and autophagy are responsible for clearing the organism of misfolded proteins, such as Aβ and tau aggregates, damaged organelles or cells, and incorrectly functioning individuals. The obtained MTDLs act as ChE, Aβ aggregation inhibitors, metal chelator agents, and ubiquitination–proteasome system (UPS) degradation pathway activators. The most promising compound from this series is compound **10** (Figure 12). Due to the apigenin-*O*-alkyl moiety, it is more potent towards *hu*AChE than *hu*BChE. The IC_50_ for *hu*AChE is equal to 360 nM, while it is 15,300 nM for *hu*BChE. The described molecule is a mixed-type blocker for *hu*AChE. In addition, molecule **10** can inhibit self-induced Aβ aggregation (IC_50_ = 5800 nM), *hu*AChE-induced Aβ aggregation, and by chelating the Cu^2+^-induced Aβ aggregation. The levels of inhibition for the last two cases are around 80% at 25 µM. This compound also possesses good antioxidant activities owing to the *O*-alkylamine side chain. Compound **10** is capable of activating the UPS system, which results in a reduced level of hyperphosphorylated tau protein and cleared APP. Apart from UPS activation, the described MTDL is capable of inducing autophagy. Finally, molecule **10** can reverse cognitive deficits and improve the dyskinesia recovery rate and efficiency response.

In 2021, Wichur et al. obtained a series of compounds based on 1-(phenylsulfonyl)-4-(piperazin-1-yl)-1*H*-indole, which were made by fusing modified benzylpiperidine (donepezil-derived fragment) with 1-(phenylsulfonyl)-4-(piperazin-1-yl)-1*H*-indole moiety [42]. In some compounds, the piperazine was replaced with an oxyethylene fragment, like in compound **11**. The derivatives act as potent BChE and Aβ aggregation inhibitors and 5-HT_6_R antagonists. The serotonergic 5-HT_6_ receptor occurs profusely in areas of the brain that are involved in learning and memory processes. Blocking this receptor can increase acetylcholine levels and reverse cognition deficits. Derivative **11** (Figure 13) was identified as the most promising due to its versatile potency towards all three targets. The 4-(2-aminoethoxy-1-(phenylsulfonyl)-1*H*-indole fragment takes part in interactions with the orthosteric binding site of 5-HT_6_R, while the 1-cyclohexylmethylamine moiety is responsible for the main interactions with BChE. The described molecule is a reversible, non-competitive inhibitor of *eq*BChE (IC_50_ = 89.9 nM) and a moderate *hu*BChE inhibitor (IC_50_ = 551.1 nM). The sp3 carbon-containing cyclohexyl group is responsible for increased activity towards *eq*BChE compared to the more rigid benzyl group. Compound **11** is also a potent antagonist of 5-HT_6_R with nanomolar values of K_i_ (4.8 nM). Compound **11** is also capable of inhibiting amyloid-beta aggregation at almost 50% at 10 µM.

In 2020, Wang et al. obtained a group of chromone–donepezil-based derivatives. The compounds were made by linking 4*H*-1-benzopyran-4-one (chromone) with the benzylpiperidine moiety of donepezil via an amide linker [43]. These derivatives are dual-acting ChE and MAO-B inhibitors. Compound **12** (Figure 14), a mixed-type *ee*AChE (IC_50_ = 370 nM) and a competitive *eq*BChE inhibitor (IC_50_ = 5240 nM), was chosen as the most promising for further evaluation. SAR studies revealed that compounds with an ethylene group in the amide linker are the most active towards both ChEs. Conversely, adding the benzyloxy substituent at position 6 in the chromone moiety leads to reduced potency toward both ChEs. Regarding MAO-B inhibition, compound **12** is a moderate, competitive, and reversible *hu*MAO-B inhibitor, with an IC_50_ value of 272 nM, and a very weak inhibitor of *hu*MAO-A, with IC_50_ = 67,200 nM. Studies of *hu*MAO-B inhibition revealed that the bulky benzyloxy substituent at position 6 increases activity towards *hu*MAO-B while adding methyl groups to the amide linker decreases *hu*MAO-B potency.

Qin et al. obtained a series of *N*-benzylpiperidine MTDLs by linking this fragment with a zinc-binding moiety, such as hydroxamic acid or o-phenylenediamine, via aromatic or aliphatic linkers [44]. These compounds act as histone deacetylase (HDAC), AChE, amyloid β aggregation inhibitors, and copper ion chelators. The most potent compound against all targets is derivative **13** (Figure 15). Inhibition of HDAC is one of the newest ways of treating AD. Studies on animal models showed that HDAC inhibitors have neuroprotective properties against AD and can help with impaired learning and memory functions [85,86,87]. Thanks to the hydroxamic acid moiety, which shows an affinity for zinc in the HDAC pocket, the compound is an efficacious inhibitor with an IC_50_ value of 170 nM (assay performed on HeLa cell nuclear extracts containing HDAC) [44]. Generally, compounds with *p*-substitution of benzene exert greater potency towards HDAC than those with *m*-substitution; however, due to the closer position of the benzene ring to the amide group than to hydroxamic acid, the *m*-substitution of the benzene in molecule **13** does not negatively affect the potency toward HDAC. Compound **13** is also a selective *ee*AChE blocker (IC_50_ = 6890 nM) thanks to the aromatic structure in the linker. More studies indicated that derivative **13** could successfully scavenge free radicals, chelate copper ions, and, because of its chelation properties, inhibit not only amyloid β self-aggregation but also Cu^2+^-induced amyloid β aggregation. The percentage inhibition of self-aggregation is about 12%, and the Cu^2+^-induced aggregation is about 34% at 20 µM. The aggregation inhibition rate for Cu^2+^-induced aggregation is three times higher than for self-aggregation for the same concentration (20 μM). The scavenging free radicals’ ability and neuroprotective properties against H_2_O_2_ are most likely due to the hydroxamic acid moiety of compound **13**.

A subsequent series of MTDLs for AD therapy was described by Malek et al. [45]. This series was obtained through a multicomponent Biginelli reaction. It combines benzylpiperidine moiety (from donepezil), modified dihydropyrimidone, and propargyloxyphenyl moiety. This series acts as ChE inhibitors, calcium channel blockers, antioxidants, and Nrf2 activators. Compound **14** was chosen for detailed evaluation (Figure 16). This compound is a moderate *hu*AChE inhibitor, with an IC_50_ value of 342 nM and an even weaker *eq*BChE inhibitor (IC_50_ = 4780 nM). SAR analysis revealed that the methylene linker between the benzylpiperidine and dihydropyrimidine central nitrogen atom is crucial for activity towards *hu*AChE. Moreover, the propargyl moiety increases the activity of the compound towards *hu*AChE compared to butynyl (present in other compounds). The short methylene linker mentioned earlier is also responsible for a higher calcium channel blockade (67% at 10 µM). Furthermore, compound **14** shows good antioxidant capacity and can induce the Nrf2 transcriptional pathway.

Another novel series of MTDLs containing a donepezil scaffold was presented by Queda et al. [46]. They obtained a series of donepezil—arylsulfonamide hybrids, which were made by linking benzylpiperidine (donepezil fragment) or its analog (benzylpiperazine moiety) with *para-*substituted aromatic sulfonamides. These compounds are dual AChE and beta-amyloid aggregation inhibitors. Compound **15** (Figure 17), the strongest *ee*AChE inhibitor (IC_50_ = 1600 nM) in the series, was chosen as the hit structure. SAR analysis showed that the short (one methylene) linker, piperidine moiety, and biphenyl group, as in compound **15**, provide the highest activity. The piperidine moiety provides a higher affinity for *ee*AChE than piperazine, and the short methyl linker results in a more rigid molecule that better fits the catalytic active site of AChE. Apart from *ee*AChE inhibition, derivative **15** can also inhibit Aβ aggregation by almost 61%, at 40 µM. The piperidine and short linker increase aggregation inhibitory activity. In addition, molecule **15** possesses a mild neuroprotective effect.

Tamaddon-Abibigloo et al. described a series of isatin–triazine–aniline hybrids [47]. These compounds were obtained by linking variously substituted anilines with isatin derivatives via triazine with a methoxy group. An isatin fragment was chosen as the donepezil indanone bioisostere. Another common motif with the donepezil structure is a benzene from the aniline fragment. The obtained molecules act as ChE inhibitors, antioxidants, and metal chelators. Compound **16** (Figure 18), a very strong *ee*AChE inhibitor (IC_50_ = 0.7 nM) and a strong *eq*BChE inhibitor (IC_50_ = 90 nM), was chosen as the lead structure. The strong ChE inhibitory potency is influenced by a substituent at the nitrogen atom of the isatin moiety. It was shown that the most active compounds contain a benzyl moiety. Moreover, the electron-donating hydroxyl group at position *orto* (in the aniline part of the molecule) is associated with strong *ee*AChE inhibitory activity. In terms of *eq*BChE, the compounds with unsubstituted aniline were the most active, but the lack of substitution negatively impacted the potency towards *ee*AChE. In addition, substitution at the *para* position of benzene in the aniline fragment decreased activity towards both ChEs. Furthermore, due to the hydroxyl group on the aniline moiety, the described compound exhibits strong free radical scavenging activity. Moreover, it possesses the ability to chelate Zn^2+^, Al^3+^, Fe^3+^, and Cu^2+^ ions due to the hydrazone moiety.

### 2.2. Tacrine-Based Multifunctional Cholinesterase Inhibitors

The first series including a tacrine scaffold is a series obtained by Rossi et al. [48]. This series of hybrids was designed by combining compounds derived from cashew nutshell liquid (CNSL), such as anacardic acid, cardanols, and cardols, with a tacrine scaffold via an eight-methylene linker. CNSL compounds are characterized by high anti-inflammatory properties. The obtained hybrids combine AChE and BChE inhibition with anti-neuroinflammatory and neuroprotective activity. Compound **17** (Figure 19), a hybrid of an anacardic acid derivative and non-modified tacrine, was chosen as the representative. It is a strong *hu*AChE inhibitor, with an IC_50_ value equal to 20.8 nM, and a highly active *hu*BChE blocker, with an outstanding subnanomolar IC_50_ value (IC_50_ = 0.0352 nM). This activity is connected with the heterodimeric structure based on the tacrine scaffold. Due to a lack of steric hindrance, compound **17** is more selective for *hu*BChE than *hu*AChE compared to its 6-chlorotacrine analog. This effect, as a result of the absence of a chlorine atom at the tacrine fragment, is observed for all pairs (unsubstituted vs. chloro derivative) of the compounds. It is worth mentioning that the presence of methyl substituents at the carboxylic and phenolic groups, leading to ester and ether formation, improves the affinity for both ChEs. Tacrine is known for its hepatotoxic side effects. Fortunately, derivative **17** did not indicate significant toxicity. Moreover, the described compound possesses anti-inflammatory activity by down-regulating interleukin-1 beta (IL-1β), cyclooxygenase-2 (COX-2), and inducible nitric oxide synthase (iNOS) and can inhibit the transcriptional activation of nuclear factor kappa-light-chain-enhancer of activated B cells (NF-κB).

The second series of novel MTDLs belonging to tacrine-based compounds are thiazolopyridyl-tetrahydroacridines, obtained by Jiang et al. [49]. The design of these compounds aimed to obtain molecules capable of targeting both ChE and tau hyperphosphorylation. This was achieved by linking a ChE inhibitor (tacrine) with a glycogen synthase kinase 3 beta (GSK-3β) inhibitor (compound **18**) (Figure 20). These hybrids were divided into two groups based on the linking process. In the first group, the tacrine moiety was linked to the thiazole side of compound **18**, while in the second group, it was linked to the pyridine side. The obtained compounds, as expected, act as AChE, BChE, and GSK-3β inhibitors. GSK-3β, one of the two isoforms of GSK-3, is a major tau kinase. It is hyperactive in AD patients, which leads to the accumulation of toxic tau aggregates in the brain. Compound **19**, which belongs to the second group of derivatives (Figure 21), was chosen as the representative. This MTDL inhibits *hu*GSK-3β (human GSK-3β), with an IC_50_ value of 22 nM, thanks to the short linker, which ensures additional hydrophobic interactions between tacrine and *hu*GSK-3β. SAR studies showed that increasing the length of the linker decreases the activity towards *hu*GSK-3β. On the other hand, the process of methylation of the carboxamide on the thiazole ring group led to the elevation of *hu*GSK-3β inhibitory activity. Compound **19** is more selective for *hu*AChE than *hu*BChE (IC_50_ = 1.2 nM and 149.8 nM, respectively). Moreover, replacing the cyclopropylmethyl group with hydrophobic groups and linker elongation are harmful to the ChE’s potency. Because of a competitive ATP fragment, 2-aminopyridine, molecule **19** was also a potent inhibitor of the dual specificity tyrosine phosphorylation-regulated kinase 1 (DYRK1: DYRK1α and DYRK1β) with IC_50_ for DYRK1α equal to 28.3 nM. DYRK1α may participate in developing the early stage of AD by phosphorylating tau and α-synuclein. In addition, hybrid **19** can reduce the phosphorylation level of the tau protein, reduce ROS generation, and ameliorate memory and cognitive decline.

In 2022, Waly et al. obtained four series of heterocyclic hybrids. The first series was obtained by linking various cyclic amines with pyrazolopyridine scaffold by amide spacer [50]. The second series was obtained by directly attaching the acridine core of tacrine with a variety of cyclic amines. The third series was obtained by linking the acridine core of tacrine with different cyclic amines by an ethylamine bridge. The last series was made by attaching a pyrazolopyridine scaffold to the amine moiety of tacrine. Such MTDLs act as ChE and Aβ aggregation inhibitors and metal chelators. Compound **20** (Figure 22), a moderate *hu*AChE (IC_50_ = 299 nM) and *hu*BChE (IC_50_ = 429 nM) inhibitor with a low selectivity index (SI = 1.43), was chosen as the compound for further studies. Structure–activity relationship analysis showed that the substitution of tacrine core at position nine with pyrazolopyridine-3-amine, as well as the introduction of the strongly electron-withdrawing nitro group at position seven, contribute to moderate ChE inhibition and strong beta-amyloid aggregation inhibitory activity (Aβ aggregation IC_50_ = 777 nM). Furthermore, hybrid **20** possesses iron, zinc, and copper chelating abilities.

Next, tacrine-based compounds were reported by Chrienova et al. The authors designed three groups of compounds [51]. The first group (A) was obtained by merging modified tacrine or tacrine moieties with two or one allyl or propargyl moieties. The second set (B) was made by linking tacrine or modified tacrine with mono or bis propargyl by a propylene linker. The third class (C) was obtained by merging a quinoline moiety with a propargyl or allyl moiety. These MTDLs were designed as both ChE and MAO-B inhibitors. Compound **21** (Figure 23), the lead structure from group A, is a moderate *hu*BChE (IC_50_ = 659 nM) and *hu*AChE (IC_50_ = 1472 nM) inhibitor. The slightly higher potency towards *hu*BChE is due to the propargyl moiety and tacrine itself. Moreover, one propargyl group instead of two was beneficial in terms of anti-ChE potency. In addition, compound **21** is able to inhibit *hu*MAO-B with an IC_50_ value of 40,390 nM due to the presence of a propargyl moiety and 7-phenoxy fragment in the tacrine core.

In 2021, Gorecki et al. presented a series of phenothiazine–tacrine heterodimers [52]. Synthesis of these heterodimers was conducted by linking variously substituted phenothiazine cores (derived from promethazine, chlorpromazine, and fluphenazine) with three different tacrine fragments (unsubstituted tacrine, 7-methoxytacrine, and 6-chlorotacrine) via an oligomethylene linker (ranging from two to five methylene groups). Phenothiazine is a motif frequently occurring in antipsychotic drugs used for the treatment of schizophrenia. On the other hand, compounds derived from the phenothiazine dye methylene blue were reported to possess anti-Aβ aggregation properties and tau filament formation inhibitory potency. Moreover, compounds containing phenothiazine can exhibit neuroprotective activity. Additionally, 7-methoxy and 6-chloro tacrines demonstrate less hepatotoxic activity than tacrine itself. The length of a linker can influence ChE inhibitory activity, making compounds more selective towards AChE or BChE. The obtained heterodimers act as ChE inhibitors and possess tau and beta-amyloid aggregation inhibitory activity. Compound **22** was chosen due to its good inhibitory activity while being relatively non-cytotoxic (Figure 24). Derivative **22** is a strong, mixed-type *hu*AChE inhibitor with an IC_50_ value equal to 8 nM and a strong *hu*BChE (IC_50_ = 190 nM) inhibitor. The stronger inhibition of *hu*AChE by compound **22** can be explained by the fact that a compound bearing a four-methylene linker can set the bulky phenothiazine moiety in a better manner toward the peripheral anionic site of *hu*AChE than compounds with shorter linkers. On the other hand, compound **22** cannot fit optimally in the broad gorge of *hu*BChE with a reduced peripheral site. Furthermore, the 6-chlorotacrine moiety in the structure of the compounds makes them more potent than tacrine towards *hu*AChE (tacrine has an IC_50_ of 3.16 nM [83]). Derivative **22** can also inhibit tau peptide (306–336) aggregation (three-repeat domain fragment). SAR analysis, in this case, revealed that shorter methylene linkers (from two to three methylene groups) on 6-chlorotacrine derivatives could increase the inhibition level in comparison to four methylene groups in compound **22**; however, the difference was not significant (the inhibition percentage is around 50% at 50 µM). The described compound can reduce not only the amount of fibrils formed but also delay the exponential phase of the aggregation. In terms of anti-aggregation properties, compound **22** can also strongly suppress Aβ-aggregation (with about 74.1% inhibition at 50 µM) due to increased ligand flexibility and a longer spacer, which allows for contact with two parallel β-sheets of Aβ.

The next series of tacrine-containing MTDLs was obtained by Fares et al. They described a group of chromene–tacrine derivatives, which were obtained by replacing the benzene ring from tacrine with a chromene scaffold [53]. These hybrids possess ChE, MAO-B, BACE1, and Aβ aggregation inhibitory activity, as well as metal chelating properties. The tacrine part is responsible for the activity toward ChE, while the chromene is involved in BACE1, MAO-B, and Aβ aggregation inhibition. Two derivatives were chosen as the most promising; however, due to the higher inhibitory activity of compound **23** towards all targets, this compound is shown (Figure 25). Its IC_50_ value for *hu*AChE is 250 nM, and for *hu*BChE is 140 nM. In the subgroup of tacrine-chromeno acetophenone derivatives, including compound **23**, the *hu*ChE inhibitory activity depends on the presence of chlorine atoms as electron-withdrawing substituents in rings A and B. Chlorine atoms in both rings A and B contribute to high *hu*AChE inhibition, while chlorine atoms in ring A ensure the elevated *hu*BChE inhibition. In the case of BACE1 (IC_50_ = 440 nM), MAO-B (IC_50_ = 2420 nM), and Aβ (IC_50_ = 740 nM) inhibition, chlorine atoms at *para* and *meta* positions of ring A also lead to the increased potency of compound **23** to these targets compared to the other compounds from the subgroup of tacrine-chromeno acetophenone derivatives. Furthermore, the described compound is able to form complexes with Fe^2+^, Cu^2+^, and Zn^2+^ ions, showing promising metal chelating properties.

The last tacrine-based MTDLs were designed, synthesized, and biologically evaluated by Long et al. [54]. They obtained capsaicin–tacrine hybrids, which were made by linking a tacrine moiety with capsaicin by an alkyl linker. The tacrine moiety ensures ChE inhibition, while the capsaicin is responsible mostly for anti-BACE1 activity. The obtained hybrids work as AChE, BChE, and BACE1 inhibitors. Compound **24** (Figure 26), which is based on hydrogenated capsaicin, displayed good potency towards all three targets (IC_50_ value for *hu*AChE is 69.8 nM, for *hu*BChE is 68 nM, and for BACE1 is 3600 nM). It is a mixed-type *hu*AChE inhibitor. According to SAR analysis, the long, seven-carbon linker is responsible for high *hu*AChE activity. Unfortunately, the long chain is not suitable for *hu*BChE inhibition, and the most potent inhibitor has a medium-length linker (from 3 to 6 carbons). Considering the BACE1 SAR analysis, the branched and long alkaneamide linker slightly decreases the activity of compound **24** towards BACE1. Finally, compound **24** can reduce cognitive impairment and memory decline.

### 2.3. Carbamate-Based Multifunctional Cholinesterase Inhibitors

The first MTDLs in this group are carbamate derivatives of *N*-salicyloyl tryptamine, obtained by Liu et al. [55]. These compounds were made using a fusion strategy, with the use of variously substituted carbamate moiety with *N*-salicyloyl tryptamine. *N*-salicyloyl tryptamine compounds were found to possess anti-neuroinflammatory properties by inhibiting the lipopolysaccharide (LPS)-induced activation of glial cells. Moreover, they also reduce the production of cyclooxygenase and have radical scavenging activity. Additionally, compounds with a carbamate moiety can exhibit higher inhibitory activity towards ChE due to the release of reversible inhibitors after transferring the carbamate moiety to enzymes. Compound **25** (Figure 27) was chosen as the lead. Surprisingly, it is a mixed-type, reversible inhibitor of both *eq*BChE (IC_50_ = 1840 nM) and *ee*AChE (IC_50_ = 3420 nM). Regarding *eq*BChE inhibition, small substituents at nitrogen atom from carbamate moiety are favorable for the effective suppression of this enzyme. Considering the anti-*eq*AChE activity, the most efficacious compounds have an unsymmetrical substitution of the mentioned carbamate group. Furthermore, the carbamate group placed in the *ortho* position is the best for obtaining optimal potency toward both ChE. Additionally, derivative **25** turned out to be effective in reducing Aβ aggregation (by 40.26% at 20 µM) and ROS production. Compound **25** also possesses anti-inflammatory properties by inhibiting interleukin-6 and tumor necrosis factor-alpha (TNF-α) production, decreasing the activity of inducible nitric oxide synthase (iNOS), and increasing interleukin-4 production (IL-4). IL-4 is involved in weakening stress-induced depression and maintaining brain homeostasis. Finally, compound **25** tends to improve learning and memory and can prevent neurodegeneration in hippocampal CA1 and CA3 regions.

The second series of MTDLs containing a carbamate moiety was described by Sang et al. in 2020 [56]. They obtained a series of *O*-carbamoyl ferulamide derivatives, which were made by fusing carbamate fragments with compound **26** (Figure 28) based on the structure of ferulic acid [88]. The purpose of this modification was to find new derivatives with high BChE inhibition and devoid of neurotoxic activity [56]. Ferulic acid is known for its free radical scavenging properties, Aβ aggregation inhibitory properties, and anti-inflammatory activity. Designed compounds possess AChE, BChE, and MAO-B inhibitory activities; the ability to reduce Aβ aggregation and tau levels; and APP clearance properties. Compound **27** (Figure 28) represents the carbamate derivatives of ferulamide and is a pseudo-irreversible, selective *hu*BChE inhibitor (IC_50_ = 970 nM, selectivity over AChE). This elevated potency towards *hu*BChE can be correlated with the presence of 1,2,3,4-tetrahydroisoquinoline as a substituent in the amide group and two alkyl substituents in the carbamate moiety. Additionally, the methoxy group of ferulic acid is crucial for maintaining *hu*BChE inhibitory activity. Regarding monoamine oxidases, compound **27** is a selective *hu*MAO-B inhibitor (IC_50_ equal to 5300 nM, selectivity over MAO-A), thanks to the 1,2,3,4-tetrahydroisoquinoline moiety and *N*-ethyl-*N*-methylcarbamate. Moreover, the methoxy group of ferulic acid elevates the *hu*MAO-B inhibitory potency. In addition, compound **27** can suppress the aggregation of Aβ (by 58.2% at 25 µM), mildly decrease neurofibrillary tangles levels, and significantly diminish APP protein levels. Last but not least, derivative **27** can improve cognitive and memory impairment by increasing brain cholinergic activity.

### 2.4. Multifunctional Cholinesterase Inhibitors with Diverse Scaffolds

In 2024, Xia et al. published a series of tryptanthrin derivatives obtained by linking the tryptanthrin core with various substituted amines via an amide linker [57]. Tryptanthrin was found to possess a broad spectrum of anti-neuroinflammatory properties, while the introduction of a side chain with a terminal amine moiety was expected to improve the activity of tryptanthrin derivatives toward AChE. The obtained series of MTDLs work as ChE inhibitors, beta-amyloid aggregation inhibitors, and copper ion chelators and have wide anti-inflammatory properties. A representative of these MTDLs is compound **28** (Figure 29), a selective and reversible *ee*AChE inhibitor (IC_50_ = 12.17 nM). The selectivity of molecule **28** can be associated with the substitution of tryptanthrin at position 2, increased length of an amide linker, and an alkylamine group with short methyl substituents. In addition, compound **28** prevents Aβ_1-42_ self-aggregation (by almost 64% at 100 µM), selectively chelates Cu^2+^ ions (with selectivity over Mg^2+^, Ca^2+^, Al^3+^, Fe^3+^, and Fe^2+^), and exhibits notable anti-neuroinflammatory properties. The described molecule inhibits the release of some cytokines (interleukin-1 beta and TNF-α) and nitric oxide, downregulates the pro-inflammatory cyclooxygenase 2 and iNOS, and protects the cells from H_2_O_2_-induced injury. Finally, compound **28** improves cognition and memory and can prevent pathological changes in the neuronal cells in the CA1 and CA3 hippocampus regions.

Another series of ChE inhibitors was obtained by Dakhlaoui et al. They designed and synthesized sulfonamide-dihydropyridine hybrids, utilizing a multicomponent reaction to combine sulfonamides with a 1,4-dihydropyridine core [58]. The 1,4-dihydropyridine is a scaffold present in the calcium channel blockers. The hydrophobic regions (aryl and alkyl groups), as well as sulfonamide groups, were introduced to ensure ChE inhibition and Nrf2 activation. Additionally, these compounds also possess antioxidant activity. Compound **29** (Figure 30), as the most promising from the series, is a dual *ee*AChE (IC_50_ = 12,600 nM) and *eq*BChE (IC_50_ = 8700 nM) inhibitor. Due to the presence of a small methyl group in the sulfonamide moiety, it is the only one from the series that is able to target *ee*AChE. Additionally, compound **29** slightly inhibits calcium channels (27% of calcium channel blockade at 10 µM), activates the Nrf2 transcriptional pathway, and possesses ROS scavenging activity.

Bhanukiran et al. designed, synthesized, and evaluated a series of 3-hydroxypyrrolidine derivatives [59]. The authors used vasicine (pyrroloquinazoline alkaloid) as a precursor for 1-(2-aminobenzyl)pyrrolidine-3-ol, which was subsequently acylated by various substituted aromatic carboxylic acids (Figure 31). The obtained compounds act as ChE and beta-amyloid aggregation inhibitors. A promising compound for further optimization is molecule **30** (Figure 31), a moderate and selective *hu*AChE inhibitor (IC_50_ = 250 nM). This enzyme preference can be linked to the presence of a bromine atom at the *para* position of the benzamide fragment. Furthermore, this compound exhibits significant free radical scavenging potency. In addition, the described derivative inhibits *hu*AChE-induced Aβ aggregation and Aβ self-aggregation, reverses deficits in memory and cognitive functions (through the cholinergic pathway), and recovers neuronal cell loss in the hippocampus region in rats. The ameliorating effect of cognitive decline was tested in a Y-maze assay performed on a scopolamine-induced memory impairment mouse model (Figure 32).

In 2023, Rao et al. published a series of α-amino phosphonate derivatives [60] obtained by combining 2,3-disubstituted quinazoline-4-one, vanillin, and α-amino phosphonates. These compounds possess ChE and beta-amyloid inhibitory activity and antioxidant properties. The combination of the mentioned structural motifs increases the activity towards beta-amyloid aggregation and ensures the ability to influence ROS generation. In addition, these MTDLs are able to nick DNA. Among the series, compound **31** (Figure 33), a moderate and selective *hu*AChE (IC_50_ = 2554 nM), was selected. The electron-withdrawing substituents (chlorine atoms) in the benzene ring and the amino phosphonate contribute positively to the *hu*AChE potency. Moreover, owing to the presence of chlorine atoms, compound **31** exerts antioxidant activity. In addition, derivative **31** prevents oxidation of DNA induced by Fenton’s reagent. Oxidation of DNA can lead to neurodegeneration, which is a crucial problem in AD. Last but not least, the presented molecule increases the conversion of toxic forms of Aβ into non-toxic ones by forming non-covalent interactions between amide or carboxylic groups of beta-amyloid and the hydrophilic end (phosphonate groups) of the derivative.

The last series of MTDLs with various structures and targeting cholinesterases as the main target was obtained by Pasieka et al. [61]. Overall, the designed compounds were based on the structure of compound **32** (Figure 34) and consist of three fragments: fragment A, fragment B, and fragment C. Fragment A has one of the following moieties: diphenylmethyl, 2,2-diphenylethyl, or 3,3-diphenylpropyl. Fragment A is connected with fragment B, which is a 1-amino-4-phenylbutan-2-ol-3-yl moiety, via amine (in the first group) or amide (in the second group) fragment. The last fragment, fragment C, is represented by various amino moieties. The obtained MTDLs act as inhibitors of BACE1, BChE, amyloid β aggregation, and γ-aminobutyric acid transporters (GATs). Compound **33** (Figure 35), a strong *eq*BChE (IC_50_ = 60 nM, noncompetitive inhibitor) and *hu*BChE (IC_50_ = 20 nM) inhibitor, was chosen as the lead structure. According to structure–activity relationship analysis, the high *eq*BChE inhibitory potency of **33** comes from the direct connection of the benzhydryl moiety with an amide group, an amide group, and benzylamine moiety as fragment C, with a bulky substituent (4-*tert*-butyl moiety). Moreover, fragments like amide group and *tert*-butyl substituent at the benzylamine moiety are involved in the *hu*BACE1 inhibition of molecule **33** (IC_50_ = 5900 nM). In terms of the GAT blockade, the crucial fragments are two or three aromatic rings connected to a polar core with a protonated nitrogen atom via an aliphatic spacer, like in compound **33**. In addition, because of the amide group, the compound exhibits inhibitory activity towards subtype 4 of *m*GAT (*m*GAT4, 55% of inhibition at 100 µM), although substituted fragment C attenuates this inhibitory activity. On the other hand, the 4-tert-butyl fragment is responsible for the elevated potency of compound **33** towards *m*GAT1 (IC_50_ = 10,960 nM) and *m*GAT2 (IC_50_ = 19,050 nM). Additionally, the described MTDL **33** can inhibit β amyloid aggregation despite bearing an amide bond, which was found to not be optimal for antiaggregatory properties.

## 3. Multifunctional H_3_ Receptor Inverse Agonists and Antagonists

**Figure 36:**
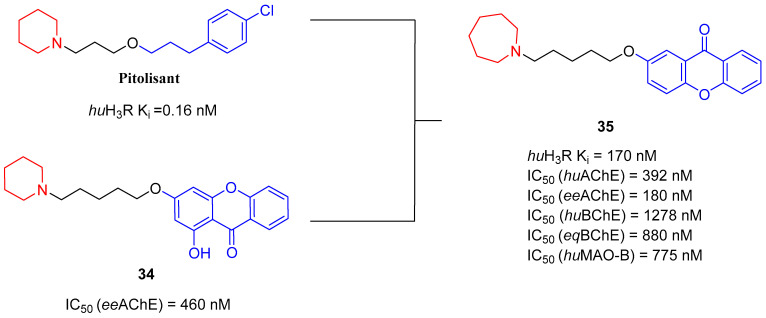
Design of compound **35** based on pitolisant and compound **34**. Colors indicate common structural motifs.

**Figure 37:**
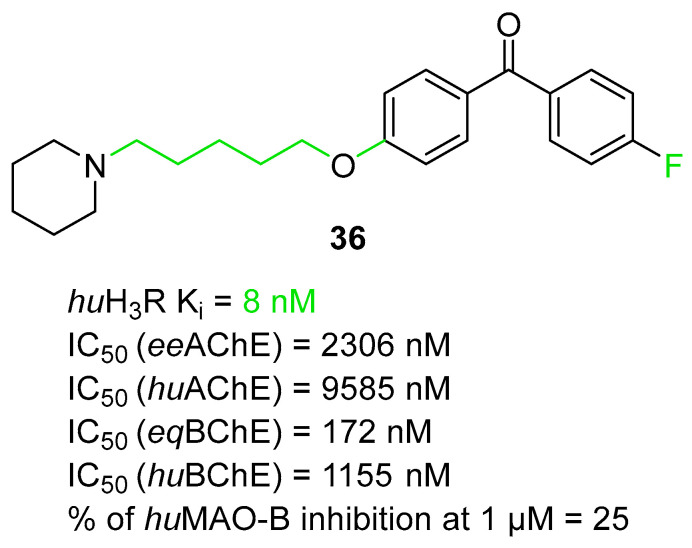
Structure of compound **36**. The green color indicates fragments that contribute to the highest huH_3_R affinity (five-methylene linker, *para* position of the alkoxyl chain, and fluorine atom).

**Figure 38:**
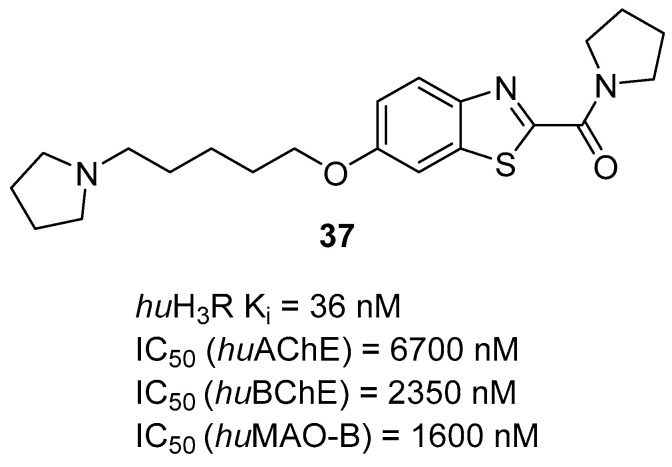
Structure of compound **37**.

**Figure 39:**
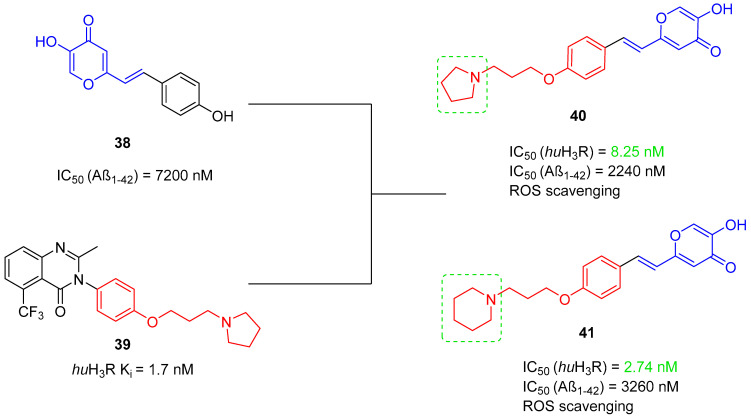
Designs of compounds **40** and **41** from molecules 38 and 39. Green shapes indicate fragments that contribute to the highest huH_3_R affinity (pyrrolidine and piperidine rings).

The histamine receptors are abundant in the area of the brain connected with memory. Blockade of these receptors is correlated with increased histamine and acetylcholine levels and improvement of cognitive functions [62]. H_3_ receptor inverse agonists/antagonists should possess three structural fragments: a tertiary basic amine linked with a bulky, lipophilic, and aromatic fragment via a linear linker [62,63]. Compounds **35**–**41** (Figure 36, Figure 37, Figure 38 and Figure 39) are representatives of this group [62,63,64,65]. In addition, compounds **35**–**37** (Figure 36, Figure 37 and Figure 38) can suppress ChE [62,63,64], and compounds **40**–**41** (Figure 39), instead of targeting ChE simultaneously with the histamine receptors, can influence beta-amyloid aggregation [65]. The most effective H_3_ receptor inverse agonists/antagonists are compounds **36** (Figure 37) (K_i_ = 8 nM) [63], **40** (Figure 39) (IC_50_ = 8.24 nM) [65], and **41** (Figure 39) (IC_50_ = 2.77 nM) [65].

The first series in this category was described by Łażewska et al., who managed to obtain xanthone-containing derivatives [62]. These compounds were designed based on the structure of a known H_3_R ligand—pitolisant [89]—and compound **34** (Figure 36), a xanthone-based cholinesterase inhibitor [62]. The authors designed a series of derivatives by linking xanthone moiety with a cyclic amine moiety by an oligomethylene linker. The obtained compounds act as histamine H_3_ receptor antagonists/inverse agonists and AChE, BChE, and MAO-B inhibitors. The representative of these MTDLs is compound **35** (Figure 36), with high *hu*H_3_R affinity (K_i_ = 170 nM), which results from the presence of azepane as a tertiary amine and a five methylene linker. Linkers longer than five methylene groups significantly decrease affinity for *hu*H_3_R. Moreover, compound **35** is a strong *ee*AChE (IC_50_ = 180 nM, noncompetitive inhibition) and a moderate *eq*BChE inhibitor (IC_50_ = 880 nM). It also inhibits *hu*AChE (IC_50_ = 392 nM) and *hu*BChE (IC_50_ = 1278 nM). Once again, azepane as an amine moiety is the most beneficial, but the length of the linker in azepane derivatives is not so important for ChE inhibition. Of note, incorporating chlorine into xanthone moiety for azepane-containing ligands generally elevates the inhibitory potencies towards both ChEs. Concerning monoamine oxidase inhibition, compound **35** tends to be a moderate, selective *hu*MAO-B inhibitor (IC_50_ = 775 nM). Finally, **35** can significantly ameliorate memory impairment; it also possesses an analgesic effect.

The second series of novel histamine H_3_ receptor ligands was presented by Godyń et al. [63]. They obtained a series of benzophenone derivatives based on compound **35** [62]. These compounds were designed by linking benzophenone moiety with a cyclic amine (methylpiperidine or azepane) by an oligomethylene linker. The obtained benzophenone derivatives act as H_3_R antagonists/inverse agonists and AChE and BChE inhibitors. The most promising for further research is compound **36** (Figure 37), with a high affinity for histamine *hu*H_3_ receptors (K_i_ = 8 nM). This high affinity is due to the *para* position of the alkoxyl chain in the benzophenone scaffold, five-methylene linker, and a fluorine atom in the benzophenone moiety. The described compound is also a non-competitive *ee*AChE (IC_50_ = 2306 nM) and a mixed-type *eq*BChE (IC_50_ = 172 nM) inhibitor. The higher potency toward *eq*BChE is observed due to the *para-*position of the alkoxyl chain in the benzophenone scaffold and the presence of a fluorine atom. In terms of *ee*AChE, the described molecule is less active because it lacks the *meta-*substitution and the six-methylene linker, both of which are preferred for higher potency. Moreover, molecule **36** can inhibit *hu*BChE (with IC_50_ = 1155 nM) and *hu*AChE (with IC_50_ = 9585 nM). Compound **36** also exhibits a slight neuroprotective effect and is able to suppress *hu*MAO-B activity by 25% at 1 µM. Unfortunately, the presented derivative has no beneficial effect on memory or learning; however, it shows significant analgesic properties.

The last series belonging to the MTDLs targeting the H_3_ receptor with ChE inhibition was obtained by Hafez et al. [64]. A benzothiazole ring was introduced as an aromatic core in the structure of compounds. A variety of alicyclic, cycloalkyl amines, and amino acids were used as the basic amine fragment, which was connected to benzothiazole via spacers consisting of two to five carbon atoms. Moreover, different esters and amides were added on the opposite side of the benzothiazole moiety. Synthesized compounds possess AChE, BChE, and MAO-B inhibitory activity and act as H_3_ antagonists/inverse agonists. Compound **37** (Figure 38) is a strong *hu*H_3_R antagonist (K_i_ = 36 nM). This property is correlated with the presence of a five-methylene linker and pyrrolidine moiety which is easily protonated in physiological pH. Moreover, compound **37** inhibits both *hu*AChE (IC_50_ = 6700 nM) and *hu*BChE (IC_50_ = 2350 nM). In terms of *hu*AChE, compounds bearing the pyrrolidine moiety are the strongest inhibitors. However, the potency of derivative **37** with the five-carbon linker is slightly weaker than that of other pyrrolidine hybrids with shorter linkers. In addition, molecule **37**, due to the presence of the pyrrolidine moiety, is also capable of inhibiting *hu*MAO-B, with the IC_50_ equal to 1600 nM.

Hu et al. described MTDLs with affinity for H_3_R but devoid of ChE inhibitory activity [65]. They obtained a series of 2-styryl-5-hydroxy-4-pyrone-based compounds by merging the 2-styryl-5-hydroxy-4-pyrone scaffold of compound **38** [90] with the phenoxypropylpyrrole moiety of molecule **39** [91] (Figure 39). The obtained compounds act as H_3_ receptor antagonists, metal ion chelators, and Aβ self-induced and Cu^2+^-induced aggregation inhibitors [65]. Compounds **40** and **41** (Figure 39) are excellent *hu*H_3_ antagonists, with IC_50_ values equal to 8.25 nM and 2.74 nM, respectively. Their high *hu*H_3_R affinity is associated with the presence of pyrrolidine and piperidine rings as amines. Moreover, both compounds can suppress self-induced beta-amyloid aggregation (IC_50_ value for compound **40** is 2240 nM, and for compound **41** is 3260 nM). In addition, derivatives **40** and **41** are good ROS scavengers. Both anti-aggregation and antioxidative properties are mainly linked with the presence of 5-hydroxy-4-pyrone. Moreover, both compounds can chelate copper and iron ions. The most important for ion chelation are hydroxy and carbonyl groups.

## 4. Multifunctional Tau and/or Beta-Amyloid Aggregation Inhibitors

**Figure 40:**
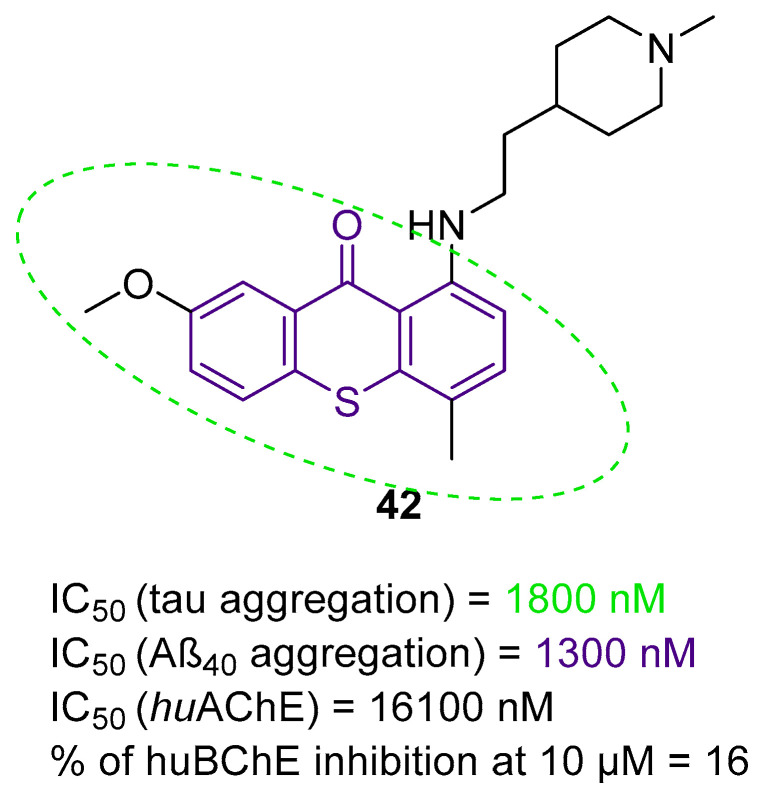
Structure of compound **42**. Green shape indicates fragments that contribute to the highest tau antiaggregating activity (thioxanthen-9-one with methoxy and methyl substituents). The violet color indicates the fragment that contributes to the highest anti-Aβ activity (thioxanthen-9-one).

**Figure 41:**
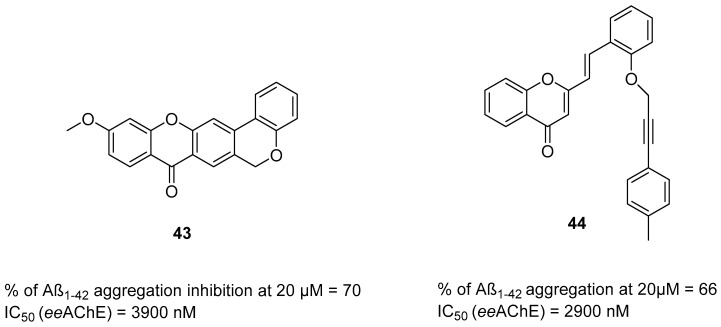
Structures of compounds **43** and **44**.

**Figure 42:**
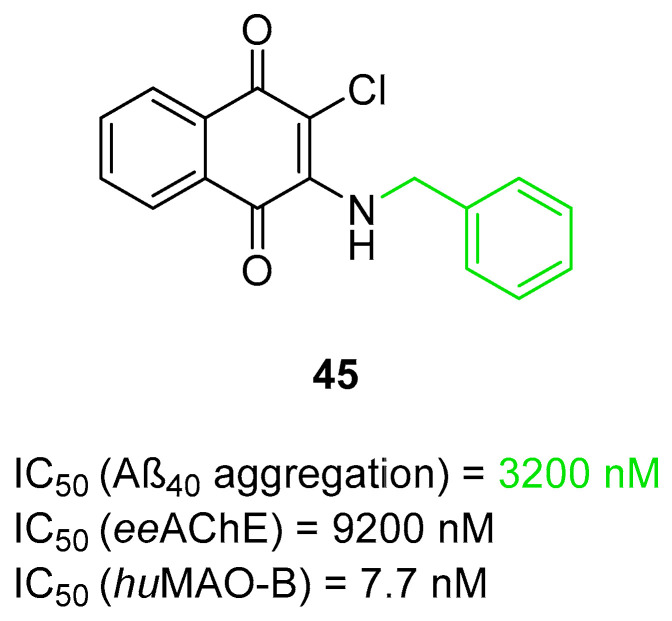
Structure of compound 45. The green color indicates the fragment that contributes to the highest anti-Aβ aggregation activity (arylalkyl fragment with one methylene group).

**Figure 43:**
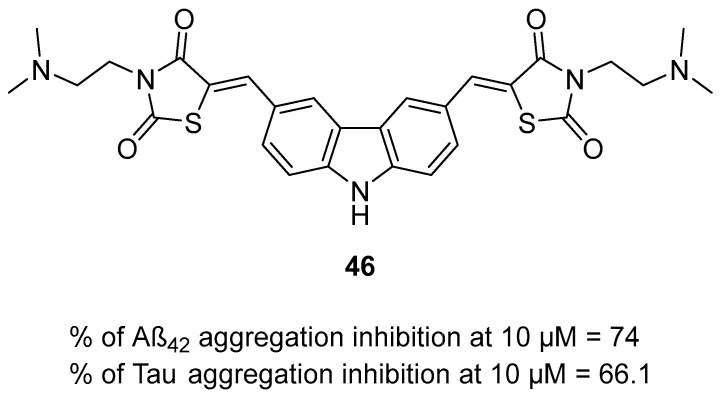
Structure of compound **46**.

**Figure 44:**
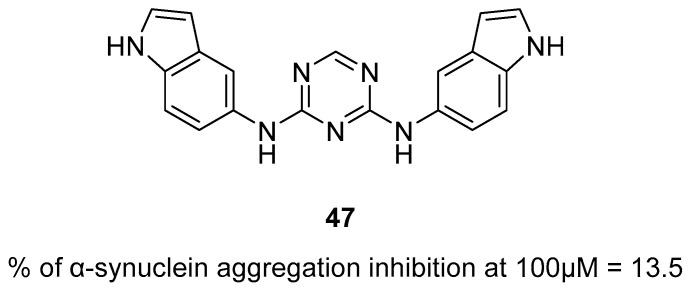
Structure of compound **47**.

**Figure 45:**
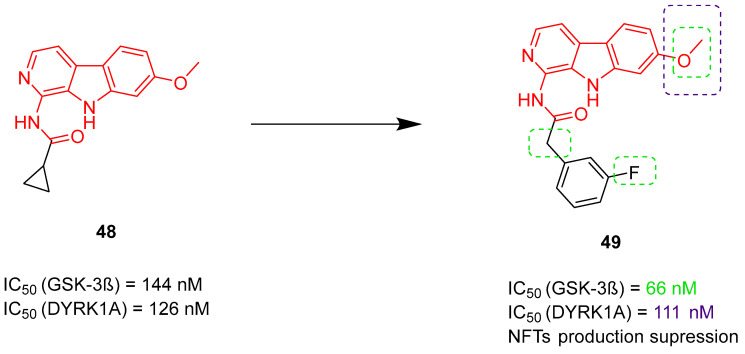
Design of compound **49** from molecule **48**. Green shapes indicate fragments that contribute to the highest GSK-3β inhibitory activity (methoxy group, fluorine atom, and methylene linker). The violet shape shows the fragment that significantly improves DYRK1A inhibitory activity (methoxy substituent).

**Figure 46:**
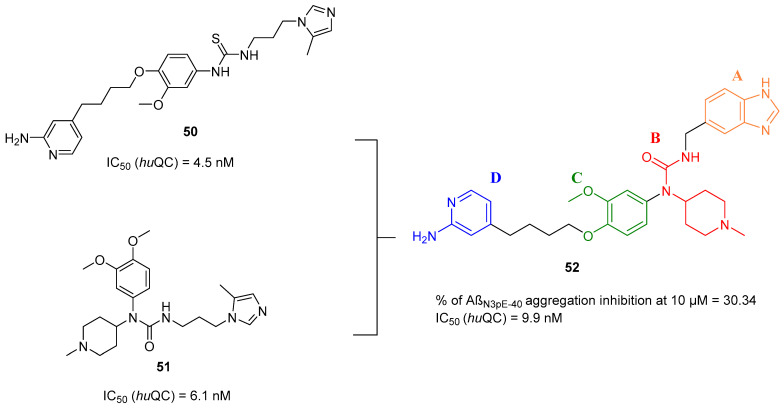
Design of compound **52** from molecules **50** and **51**. Colors represent the main pharmacophoric features.

The beta-amyloid plaques and neurofibrillary tangles accumulate, which leads to inflammation in the brain and can cause cell dysfunction or even cell death [42]. Antiaggregating properties, direct or indirect, were shown for compounds **42**–**47**, **49** and **52** (Figure 40, Figure 41, Figure 42, Figure 43, Figure 44, Figure 45 and Figure 46). Compounds **42** (Figure 40) (IC_50_ for tau aggregation = 1800 nM and for beta-amyloid aggregation = 1300 nM) [66], **45** (Figure 42) (IC_50_ for Aβ = 3200 nM) [68], **46** (Figure 43) (% inhibition for tau aggregation = 74% and for Aβ = 66.1% at 10 µM) [69], and **47** (Figure 44) can suppress the formation of Aβ and hyperphosphorylated tau aggregates [70]. On the other hand, molecules **43** (Figure 41) (% inhibition of Aβ aggregation = 70% at 20 µM) [67], **44** (Figure 41) (% inhibition of Aβ aggregation = 66% at 20 µM) [67], and **52** (Figure 46) (% of Aβ aggregation = 30.34 at 10 µM) influence only the aggregation of beta-amyloid [72]. Compound **49** (Figure 45) is associated with lowering the level of tau protein hyperphosphorylation by inhibiting glycogen synthase kinase-3 beta (GSK-3β) (IC_50_ = 66 nM) and dual specificity tyrosine phosphorylation regulated kinase 1A (DYRK1A) (IC_50_ = 111 nM) [71]. The best anti-aggregation properties are presented by molecules **42** [66], 45 [68], and 49 [71].

The first MTDLs with antiaggregating properties are xanthen-9-one-based derivatives obtained by Tonelli et al. [66]. These compounds were designed by linking thioxanthen-9-one or xanthen-9-one with quinolizidine, *N*-methylpiperidine, or *N,N*-ethylamine moiety via various alkyl linkers. The obtained compounds act as Aβ and tau aggregation inhibitors and AChE and BChE inhibitors and possess neuroprotective properties. Compound **42** (Figure 40) shows outstanding activity in inhibiting tau protein and Aβ amyloid aggregation. Due to the electron-donating methoxy group at position seven of the thioxanthone moiety, as well as the methyl group at position four, derivative **42** can significantly suppress tau protein aggregation (IC_50_ = 1800 nM). Of note, the separation of two nitrogen atoms by five carbon atoms is crucial for high tau inhibition. Additionally, the thioxanthen-9-one scaffold is more effective than xanthen-9-one in terms of Aβ (IC_50_ = 1300 nM) and tau aggregation suppression. Compound **42**, in addition to inhibiting tau aggregation, also protects against tau-induced toxicity. Regarding ChE inhibition, derivative **42** is a very weak *hu*AChE (IC_50_ value is 16,100 nM) and *hu*BChE inhibitor (16% of inhibition at 10 µM). SAR analysis revealed that the conversion of the quinolizidine ring (from the most potent *hu*BChE inhibitor) to *N*-methylpiperidine (compound **42**) notably reduced affinity for *hu*BChE. Unfortunately, compound **42** has high cytotoxicity at concentrations close to the IC_50_ values for tau and Aβ aggregation inhibition. This indicates a narrow safety margin.

The second series of aggregates targeting MTDLs, chromeno[3,4-*b*]xanthones and (*E*)-2-[2-(propargyloxy)styryl]chromones, were obtained by Malafaia et al. [67]. These compounds were obtained by the fusion of different chromone and xanthone scaffolds. Both series act as dual AChE and Aβ aggregation inhibitors. The described molecules can significantly influence the process of beta-amyloid aggregation due to the highly flat, aromatic, and hydrophobic scaffolds, as well as hydrogen bonding spots such as methoxy groups. Among all obtained derivatives, compounds **43** from chromeno[3,4-*b*]xanthones and **44** from (E)-2-[2-(propargyloxy)styryl]chromones (Figure 41) were chosen as the most promising. Both compounds are moderate *ee*AChE inhibitors, but overall, the rigid chromeno[3,4-*b*]xanthones has a higher potency for *ee*AChE. Compound **43** (IC_50_ = 3900 nM) also owes its higher potency to the presence of a methoxy group at position 11. For the series with compound **44**, the important fragment for anti-AChE activity is the terminal triple bond with an attached benzene ring (IC_50_ = 2900 µM). Moreover, both compounds possess the ability to delay the nucleation phase, which is the first step in the Aβ aggregation process. SAR analysis of the series with compound **44** revealed that the presence of a benzene ring attached to a triple bond positively influenced Aβ aggregation inhibition ability (66% of beta-amyloid aggregation suppression at 20 µM). In comparison to compound **44**, compound **43** is a more effective inhibitor of Aβ aggregation due to the flat aromatic moiety and the presence of the methoxy group (70% of beta-amyloid aggregation suppression at 20 µM).

The next series of MTDLs was described by Campora et al. They obtained a series of naphthoquinone and anthraquinone derivatives linked with benzene, indole, benzimidazole, or benzotriazole moieties via an alkyl chain [68]. These new compounds act as Aβ aggregation, PHF_6_ tau fragment (a hexapeptide fragment of the protein tau, part of the binding region and prone to aggregation [92]), AChE, and MAO-B inhibitors. Compound **45** (Figure 42), which is a naphthoquinone derivative, has the ability to suppress the formation of Aβ aggregates (IC_50_ = 3200 nM). This significant effect is linked to the short one-methylene linker and the arylalkyl substituent in the amine group. In general, the more nonpolar side chain, the higher the Aβ aggregation inhibitory activity was observed. In addition, molecule **45** is a strong reversible *hu*MAO-B inhibitor (IC_50_ = 7.7 nM) due to the presence of the *N*-arylalkyl group. Moreover, compound **45** selectively inhibits *ee*AChE (IC_50_ = 9200 nM). While the nonpolar side chain improves the compound’s beta-amyloid antiaggregating potency, it negatively impacts *ee*AChE inhibitory activity. Apart from enzyme-inhibiting properties, molecule **45** also possesses the capacity to inhibit tau aggregation.

In 2022, Gandini et al. described a series of bivalent compounds bearing 2,4-thiazolidinedione scaffolds as amyloid peptide recognition motifs [69]. These bivalent compounds were obtained by linking two identical substituted 2,4-thiazolidinedione cores by variable aromatic linkers. The described bivalent agents act as dual tau and Aβ aggregation inhibitors. The planar, highly π-conjugated structures used for linkers are known for interacting with aggregates via π-π stacking and van der Waals interactions. On the other hand, the 2,4-thiazolidinedione scaffold is known for tight binding with tau aggregates and disrupting oligomers. To provide additional electrostatic interactions with aggregates, ionizable groups, such as tertiary amines, are introduced in the ligand structures. Compound **46** (Figure 43), which can decrease the aggregation of both Aβ and tau by about 70% at 10 µM, was chosen as the representative. These two anti-aggregatory properties can be connected with a carbazole linker and decorated with an ionizable *N,N*-dimethylaminoethyl 2,4-thiazolidinedione scaffold. Moreover, the tertiary amine is preferable to carboxylic acid as an ionizable fragment. In addition, the substituent at the nitrogen atom of the 2,4-thiazolidinedione scaffold is important for reducing cytotoxicity. According to in vivo studies, compound **46** can reduce Aβ levels in Aβ_42_ *Drosophila* adult brains by up to 80%, contributing to increased life span and locomotive ability in this AD animal model.

Ramirez et al., in 2023, described a series of *N*- and *O*-linked indole triazines, which were obtained by linking triazine and indole moieties via nitrogen or oxygen atoms [70]. The compounds possess α-synuclein, tau, and Aβ aggregation-suppressing properties. Derivative **47** is the most active compound obtained in this group; hence, it is described further (Figure 44). Starting with α-synuclein aggregation, compound **47**, containing a nitrogen linker, exhibits almost 14% inhibition, which is the highest among the values of all the compounds. Compound **47** can also prevent tau 2N4R isoform oligomerization in a dose-dependent manner. Finally, molecule **47** can impair beta-amyloid aggregate formation.

The next series of MTDLs targeting the formation of the aggregates are harmine derivatives obtained by Qiu et al. [71]. Their structures are based on compound **48** (Figure 45), described previously [93]. The authors introduced aliphatic and aromatic rings with different linker lengths at position one of β-carboline and substituted the benzene ring with various groups [71]. The obtained derivatives act as dual inhibitors of glycogen synthase kinase-3 beta (GSK-3β) and dual specificity tyrosine phosphorylation regulated kinase 1A (DYRK1A). These enzymes are the key kinases for tau phosphorylation. By inhibiting them, it is possible to stop the hyperphosphorylation process and reduce NFT formation. A representative of these MTDLs is compound **49** (Figure 45), a potent GSK-3β (IC_50_ = 66 nM) and DYRK1 (IC_50_ = 111 nM) inhibitor. It turns out that the methoxy group at position seven is important for maintaining potency towards both of the enzymes. Moreover, the most favorable for high GSK-3β inhibition are one fluorine atom (preferably at *position* meta) and a short amide linker at position one of the β-carboline. In addition, increasing the number of fluorine atoms decreased the potency for GSK-3β. Last but not least, the described dual inhibitor significantly decreases NFT’s production in a concentration-dependent manner.

Van Manh et al. designed, synthesized, and evaluated a series of *N*’-substituted *N*-(4-aminoalkylphenyl)thiourea/urea compounds [72]. These derivatives were obtained by combining potent glutaminyl cyclase inhibitors (compounds **50** and **51**), which were obtained earlier by the authors (Figure 46) [94,95]. Novel MTDLs contain four main pharmacophores: a zinc-binding group (A), a hydrogen bond donor (B), a phenyl group (C), and an Arg-mimicking group (D). The obtained compounds inhibit glutaminyl cyclase and Aβ_N3pE-40_ aggregation [72]. Glutaminyl cyclase (QC) catalyzes the conversion of the *N*-truncated Aβ peptide (Aβ_3-40/42_), which is obtained from Aβ_40/42_, into the pyroglutamate peptide Aβ_N3pE-40/42_, and its levels are elevated in the AD brain [96,97,98]. Aβ_N3pE_ peptide is a constituent of Aβ plaques, which are formed in the early stage of AD [96]. Compound **52** (Figure 46) is a potent *hu*QC inhibitor (IC_50_ = 9.9 nM) [72]. Most compounds in these series bear the imidazole group as a zinc-binding agent. However, this group is known for causing cytotoxicity due to hERG and cytochrome P450 inhibition. Because of this, the benzimidazole group is more desired as a zinc-binding agent despite its lower potency towards human glutaminyl cyclase. SAR analysis revealed that attaching bulky 1-methylpiperidine to urea in compound **52** contributes to elevated activity. In vivo studies of compound **52** show that it suppresses Aβ_N3pE-40_ (30.34% aggregation inhibition at 10 µM via intracerebroventricular injection) and Aβ_N3pE-42_ formation. It also decreases Aβ_40_ and Aβ_42_ levels and can restore impaired memory in mice.

## 5. Multifunctional MAO-B Inhibitors

**Figure 47:**
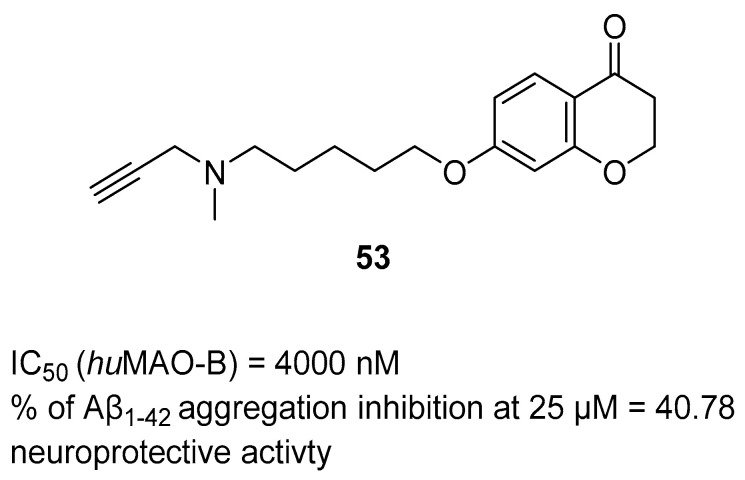
Structure of compound **53**.

**Figure 48:**
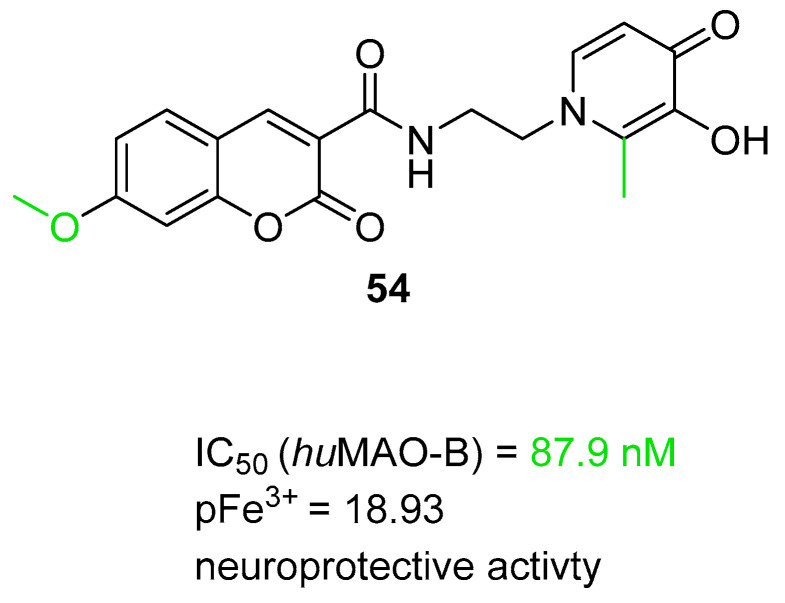
Structure of compound **54**. The green color indicates fragments that contribute to the highest *hu*MAO-B inhibitory activity (methyl substituent at 3-hydroxypyridin-4-one and methoxy group at position 7 of the coumarin scaffold).

While MAO-A is associated with depression, MAO-B levels increase with age and are associated with increased levels of oxidative free radicals, dysfunction of cholinergic neurons, and the formation of amyloid plaques [73]. In this section, we present two MAO-B inhibitors, compound **53** (Figure 47) [73] and compound **54** (Figure 48) [74]. Derivative **53** can also influence beta-amyloid, and molecule **54** is a potent iron chelator.

MTDLs targeting MAO-B were obtained by Xie et al. [73]. Inspired by the selective and irreversible MAO-B inhibitor rasagiline (IC_50_ = 141.7 nM [73]) and the selective and irreversible MAO-A inhibitor clorgyline (IC_50_ = 4.58 nM [73]), they obtained new compounds by combining clorgyline’s fragment containing *N*-propargyl amine with an optimized 2,3-dihydro-1*H*-indene rasagiline scaffold. These derivatives act as MAO-B and Aβ amyloid aggregation inhibitors. A representative of this series is compound **53** (Figure 47), a selective, competitive, and reversible *hu*MAO-B blocker (IC_50_ = 4000 nM). The significant contribution to its activity can be assigned to the five methylene units in the ether linker between the benzene ring and *N*-propargyl amine moiety (increasing the linker length further causes a decrease in activity) and replacing the 2,3-dihydro-1*H*-indene scaffold with a chromanone scaffold. Additionally, molecule **53** has good Aβ self-induced aggregation inhibitory activity (almost 50% aggregation suppression at 25 µM) and neuroprotective activity.

Jiang et al. obtained a series of (3-hydroxypyridin-4-one)-coumarin hybrids by linking substituted coumarin core with a modified 3-hydroxypyridin-4-one via an amide linker [74]. The coumarin scaffold provides MAO-B inhibitory activity, while 3-hydroxypyridin-4-one is a known iron chelator. Compound **54** (Figure 48) was chosen as the representative due to its high *hu*MAO-B potency (IC_50_ = 87.9 nM) and very low cytotoxicity. The 2-methyl substituent at 3-hydroxypyridin-4-one moiety and the methoxy substituent at the C_7_ atom of the coumarin scaffold significantly contribute to the compound’s potency. Apart from the MAO-B inhibitory activity, compound **54** possesses excellent Fe^3+^ chelating properties and cytoprotective activity against Aβ_1-42_ and ROS and can significantly ameliorate cognition impairment.

## 6. Multifunctional Metal Chelators and Reactive Oxygen Species Scavengers

**Figure 49:**
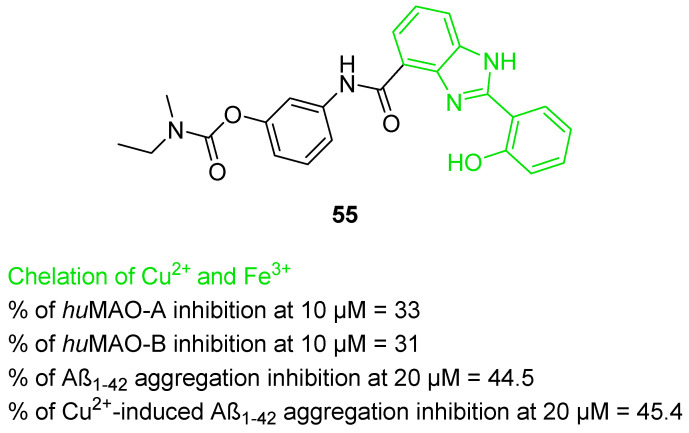
Structure of compound **55**. The green color indicates the fragment that contributes to the metal chelating properties (hydroxyphenylbenzimidazole).

**Figure 50:**
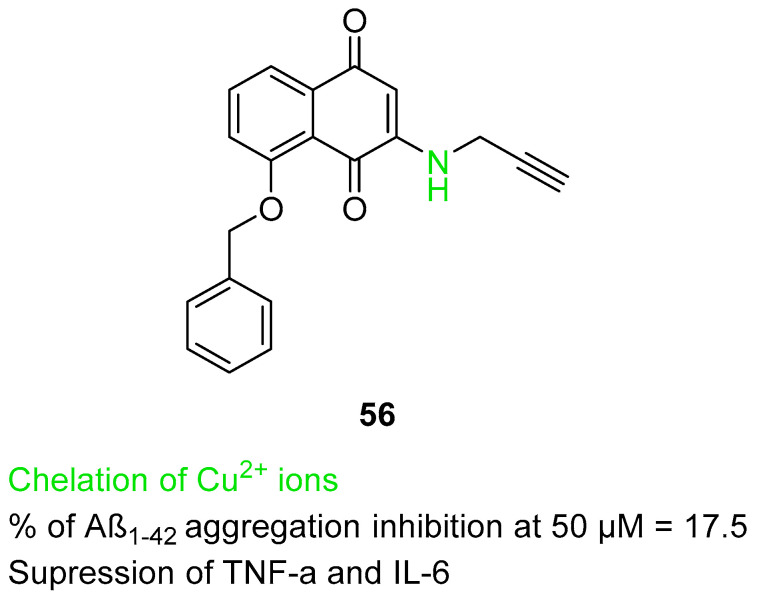
Structure of compound **56**. The green color indicates the fragment that contributes to the metal chelating activity (amine group).

**Figure 51:**
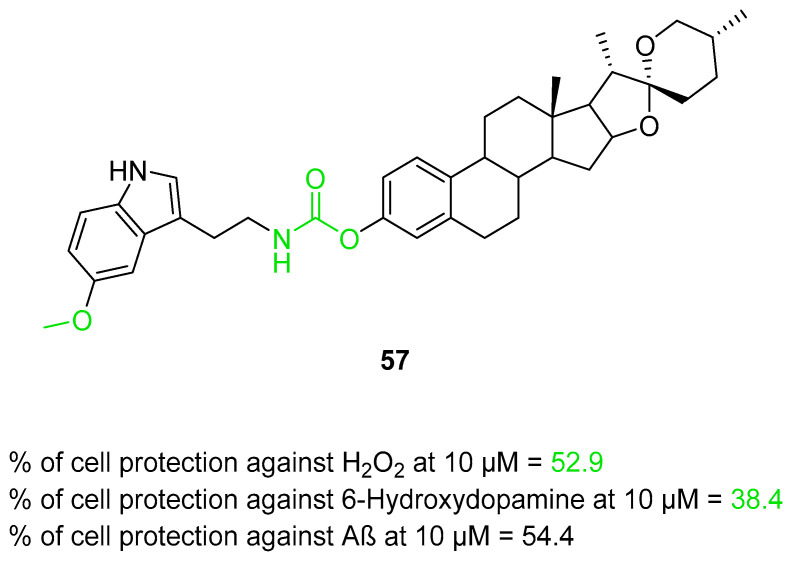
Structure of compound **57**. The carbamate group and methoxy substituent at the indole moiety (highlighted in green) are involved in the significant antioxidant activity of the compound.

The most important in metal dyshomeostasis in the AD brain are copper and iron ions. The copper ions can also contribute to beta-amyloid plaque formation [13]. On the other hand, ROS are an important factor in inflammation and neuron damage in the AD brain [99]. Two MTDLs, **55** (Figure 49) [75] and **56** (Figure 50), are the representatives of metal ion chelators due to the presence of atoms with lone electron pairs (oxygen and nitrogen), which can coordinate the metal ions [76]. Compound **57** (Figure 51) is an example of an antioxidant with protective properties against beta-amyloid toxicity, H_2_O_2_, and 6-hydroxydopamine [77].

The first series of MTDLs with metal ion chelating properties are rivastigmine–benzimidazole hybrids obtained by Vincente-Zurdo et al. [75]. These hybrids were obtained by linking the rivastigmine core with hydroxyphenylbenzimidazole via an amide linker. These compounds act as metal chelators, copper-induced Aβ aggregation inhibitors, and MAO inhibitors (MAO-A and MAO-B). In this series, compound **55** (Figure 49) shows very good metal ion chelating properties. The hydroxyphenylbenzimidazole moiety is responsible for biometal chelation. Coordination occurs through the phenolic oxygen atom and the nitrogen (N3) atom of the imidazole ring. Compound **55** possesses the ability to coordinate Fe^3+^ and Cu^2+^ metal ions with stronger binding to the second ion. This preference can be explained by the involvement of the carbonyl oxygen from the linker in the coordination. Moreover, hybrid **55** can inhibit Aβ self-aggregation (by 44.5% at 20 µM) and Cu^2+^-induced Aβ aggregation (by 45.4% at 20 µM). Because the level of inhibition of Cu^2+^-induced aggregation is only slightly higher, the aggregation suppression of Aβ must be regulated by the ability of the compound to intercalate between β-sheets of Aβ fibrils. In addition, the described MTDL can block human MAOs weakly. Compound **55** only establishes one hydrogen bond with the residues of *hu*MAO-A’s active site; hence, due to the lack of more stabilizing interactions on the active side of the enzyme, compound **55** is not very potent toward *hu*MAO-A. It has an enzyme inhibition level of 33% at 10 µM. Regarding *hu*MAO-B, compound **55** is involved in two π-π stacking interactions and hydrogen bonding with residues in *hu*MAO-B’s active site. Due to these, it is more selective for *hu*MAO-B, with inhibition of 31% at 10 µM.

The next series was described by Mezeiova et al. They obtained a series of compounds by decorating the naphthoquinone core with moieties associated with MAO inhibition, such as *N*-propragylamine or *N*-methyl-*N*-propargylamine fragments [76]. The obtained MTDLs act as MAO and amyloid-beta aggregation inhibitors, ROS scavengers, and metal chelators. The authors identified three compounds; however, due to the high cytotoxicity of two of them, compound **56** (Figure 50) was selected as the representative. Regarding metal complexation, the presented MTDL forms aggregated species with Cu^2+^ ions, and the Cu^2+^ ions are captured by the amine group. The same group is also responsible for interaction with radical species in the presence of Cu^2+^ ions. In terms of Aβ aggregation inhibition, compound **56** can suppress the aggregation process mildly (by 17.5% at 50 µM). This may be the result of a lack of methyl group at the nitrogen atom of the *N*-propargylamine moiety. Derivatives bearing the *N*-methyl group have a higher inhibition level. In addition, compound **56** has anti-inflammatory properties, decreasing TNF-α and IL-6.

In 2021, Zhou et al. designed, synthesized, and evaluated a series of diosgenin–indole derivatives where both of these cores are linked via an ester or carbamate linker [77]. The obtained molecules act as antioxidants, ROS scavengers, and neuroprotective agents against Aβ-induced toxicity. The representative for this group is compound **57** (Figure 51), a good antioxidant. This property results from a carbamate linker and a methoxy group acting as an electron-donating substituent at the indole moiety (around 50% protection of cells against H_2_O_2_ and about 40% against 6-hydroxydopamine at 10 µM). The same structural features are responsible for the significant neuroprotective activity against Aβ aggregates (54.4% protection of cells at 10 µM). Moreover, the described compound can significantly ameliorate memory and learning ability.

## 7. Multifunctional BACE1 Inhibitors

**Figure 52:**
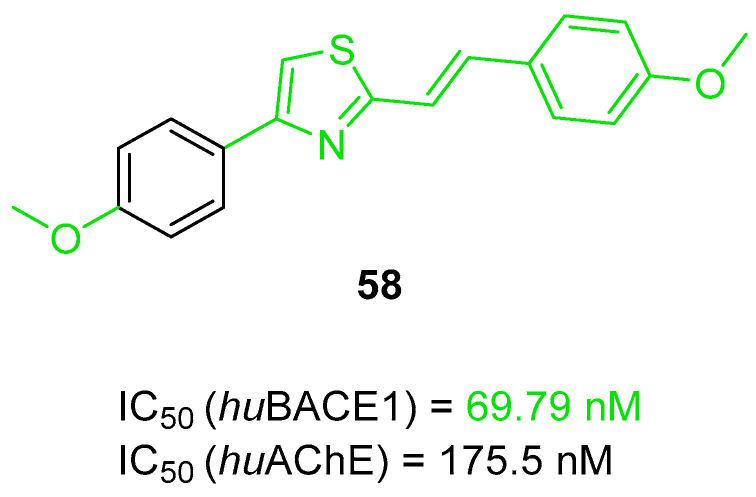
Structure of compound **58**. The green color indicates fragments that contribute to the highest *hu*BACE1 activity (methoxy groups and styryl-thiazole core).

**Figure 53:**
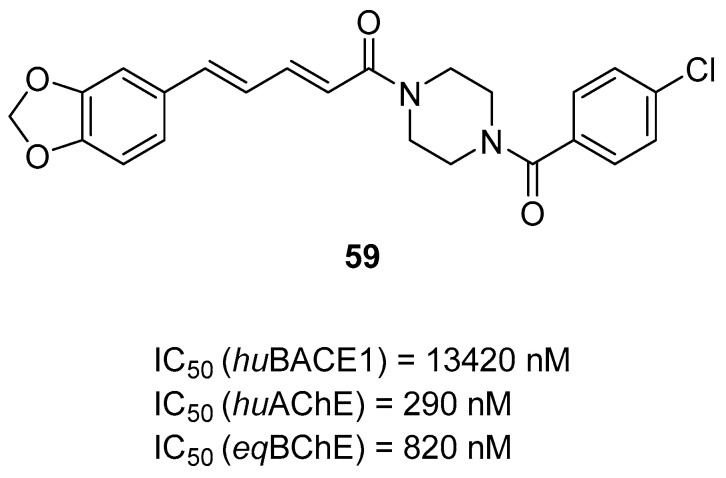
Structure of compound **59**.

**Figure 54:**
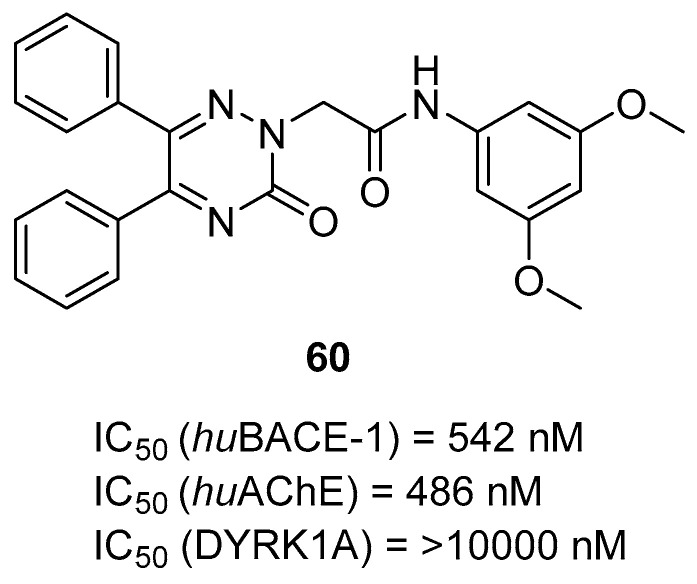
Structure of compound **60**.

The BACE1 enzyme is an important factor in the formation of the insoluble amyloid plaques from APP [79]. In this section, we present compounds **58** (Figure 52) [78], **59** (Figure 53) [79], and **60** (Figure 54) [80] with BACE1 inhibitory activity. Additional properties of the mentioned derivatives are the influence on cholinergic transmission by blockade of ChE and prevention of beta-amyloid plaque formation.

In 2023, Gouleni et al. described a series of styryl-thiazole hybrids, which were obtained by fusing the thiazole ring with a cinnamic acid [78]. The derived hybrids act as Aβ-aggregation, BACE1, and AChE inhibitors. The thiazole pharmacophore is involved in AChE inhibition, while the cinnamic acid is known for its anti-inflammatory and antioxidant properties. The most versatile towards all targets among the styryl-thiazole hybrids is compound **58** (Figure 52), a strong *hu*BACE1 (IC_50_ = 69.79 nM) inhibitor. Due to the lack of electron-withdrawing groups at aromatic rings, molecule **58** is not maximally potent in terms of *hu*BACE1 inhibition, but it can simultaneously inhibit *hu*AChE (IC_50_ is 175.5 nM) and decrease Aβ aggregation by about 80%. This multi-targeting capability of this compound is improved by two electron-donating methoxy groups attached to both aromatic rings. Last but not least, the described MTDL can penetrate BBB, although more poorly compared with other described compounds.

Bhanukiran et al. described a series of piperine-based MTDLs [79]. The design of this series embraced the conversion of piperine to piperic acid. The carboxylic group of piperic acid was then condensed with various substituted and unsubstituted cyclic amines. The described compounds act as ChE, BACE1, and beta-amyloid aggregation inhibitors. Ligand **59** (Figure 53) was chosen as the representative due to its ability to suppress *hu*BACE1, with an IC_50_ value equal to 13,420 nM. In addition, compound **59** is a moderate *hu*AChE (IC_50_ = 290 nM) and *eq*BChE (IC_50_ = 820 nM) inhibitor. This ChE inhibitory activity results from the 1,4-disubstituted piperazine ring, the presence of the carbonyl groups, and the chlorine atom as a substituent in the benzene ring. In addition, molecule **59** suppresses Aβ self-induced aggregation and AChE-induced Aβ aggregation and also possesses antioxidant activity. Moreover, compound **59** has the capacity to enhance cognition and memory, mostly by inhibiting AChE in the brain. The ability to improve memory was tested in a Y-maze assay in a scopolamine-induced memory-impaired mouse (Figure 32).

Another series of MTDLs targeting BACE1 was reported by Waiker et al. [80]. The authors obtained a series of compounds bearing a 5,6-diphenyl-1,2,4-triazin-3(2*H*)-one scaffold. These derivatives act as AChE, DYRK1A, huBACE1, and beta-amyloid aggregation inhibitors. Molecule **60** (Figure 54), a mixed-type, non-competitive *hu*AChE inhibitor (IC_50_ = 486 nM), moderate *hu*BACE1 inhibitor (IC_50_ = 542 nM), and weak DYRK1A inhibitor (IC_50_ > 10,000 nM) was chosen as the lead structure. Weak DYRK1A potency can be associated with a lack of electron-withdrawing substituents (the only compound with significant DYRK1A potency possesses a chlorine atom and a nitro group as substituents) on the *N*-phenylacetamide moiety. On the other hand, two strong electron-donating substituents on the *N*-phenylacetamide fragment are responsible for the good inhibitory potency for both *hu*BACE1 and *hu*AChE. However, increasing the number of methoxy substituents from two to three negatively influences the inhibitory activity towards both enzymes. Moreover, compound **60** can decrease *hu*AChE-induced and self-induced Aβ aggregation; this was tested in a thioflavin-T assay. Aβ self-aggregation was inhibited in the range of 23.6–68.5% for 5-20 µM of compound **60**, while for *hu*AChE-induced, it was 41.3–81.1% for the same concentration range. In terms of animal studies, molecule **60** restored memory deficits in scopolamine-treated mice and decreased the levels of BACE1 and beta-amyloid in rat hippocampal regions. The ability to improve cognitive decline was tested in a Y-maze assay on a scopolamine-induced memory-impaired mouse (Figure 32).

## 8. Multifunctional NMDAR Blockers

**Figure 55:**
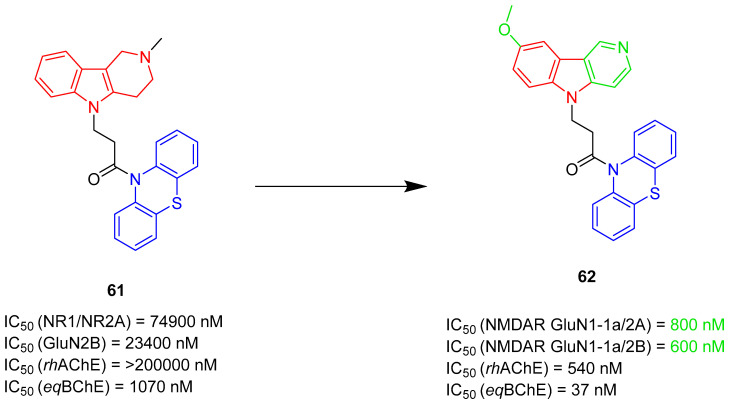
Design of compound **62** from compound **61**. Methoxy substituent at β-carboline and complete aromatization (colored in green) contribute to increased NMDAR inhibitory activity.

**Figure 56:**
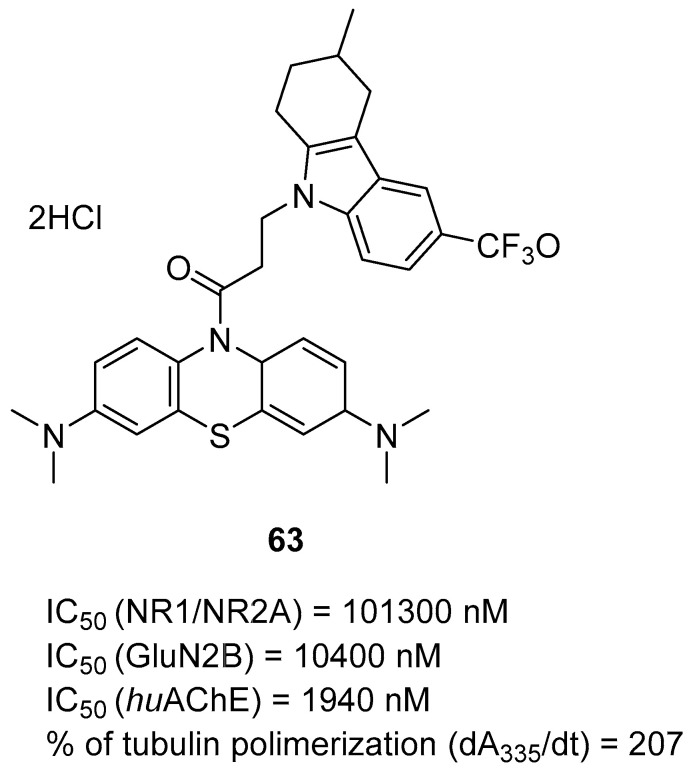
Structure of compound **63**.

NMDA receptors are an important factor in synaptic plasticity. However, excessive NMDAR activity can cause cell death, which can be one of the underlying causes of Alzheimer’s disease [100]. In this paragraph, we present compounds **62** (Figure 55) [81] and **63** (Figure 56) [82], NMDA receptor antagonists. Both of the compounds bear the scaffold from the phenothiazine family: compound **62** contains phenothiazine, and compound **63** contains methylene blue, which has an affinity for NMDAR. In addition, both of the compounds are ChE inhibitors, and compound **62** can also inhibit beta-amyloid aggregation, while compound **63** possesses neuroprotective properties and can stabilize the structure of microtubules.

The first series of NMDAR blockers was obtained by Schwarthoff et al. [81]. They described a series of γ-carboline–phenothiazine derivatives, which were designed based on compound **61** (Figure 55) [101]. The authors designed this series by changing the carboline moiety by adding various substituents into the benzene fragment [81]. In addition to the influence on NMDAR, the obtained compounds can also inhibit ChE. Molecule **62** (Figure 55), a moderate NMDAR blocker (IC_50_ (NMDAR GluN1-1a/2A) = 800 nM and IC_50_ (NMDAR GluN1-1a/2B) = 600 nM) was chosen as the lead. Conventional NMDARs consist of two glycine-binding GluN1 and two glutamate-binding GluN2 subunits [102]. In AD-affected areas of the brain, the most widely occurring subunits are GluN2A and GluN2B [103]. SAR analysis revealed that to design a potent γ-carboline-based NMDA receptor blocker, the complete aromatization of carboline moiety is obligatory. Moreover, electron-donating substituents at position eight of carboline moiety are responsible for stronger NMDA receptor-blocking activity. Furthermore, the described derivative can influence both ChEs, although it is more selective towards BChE. This certain preference for *eq*BChE (IC_50_ = 37 nM) over rhinoceros acetylcholinesterase (*rh*AChE) (IC_50_ = 540 nM) results from the bulky structure of the phenothiazine moiety.

The next series of antagonists was obtained by Bachurin et al. [82]. They presented a series of methylene blue-based derivatives, which were obtained by linking the methylene blue scaffold with cycloalkaneindoles via 1-oxopropylene spacer. The obtained compounds act as NMDAR blockers and AChE-induced Aβ aggregation inhibitors. In addition, they inhibit AChE. Compound **63** (Figure 56), a weak NMDAR antagonist (IC_50_ (NR1/NR2A) = 101,300 nM and IC_50_ (GluN2B) = 10,400 nM) was chosen as the representative. This MTDL is a reversible *huA*ChE inhibitor (IC_50_ = 1940 nM). The strong electron-withdrawing group (CF_3_O) in the indole core significantly contributes to the compound’s potency. In addition, compound **63** can decrease ROS generation, stabilize the structure of tau microtubules by stimulating polymerization, and influence mitochondrial functions. Regarding the influence on mitochondria, compound **63** can mitigate the activity of complex I inhibitors and stimulate respiratory chain activity. It also has a neuroprotective effect by preventing calcium-induced mitochondrial depolarization.

## 9. Summary

Currently, there is no effective treatment for Alzheimer’s disease available for patients. The available medicines are effective only at slowing the progression of AD and alleviating symptoms. Although new drugs are being developed, the majority of them are terminated before or during clinical trials. In medicinal chemistry, the concept of creating multi-target-directed ligands targeting underlying causes of diseases with complex pathological mechanisms such as AD has recently been considered the most effective approach. The majority of the compounds are based on the structures of drugs that are commonly used for AD, such as donepezil, or those that were used in the past, like tacrine. This way, newly designed compounds retain the activity/properties of the original compounds and, due to structural optimization, are more druggable and can influence new targets. One donepezil-based MTDL, compound **1** (Figure 5), is exceptionally noteworthy due to its higher potency toward *hu*AChE (IC_50_ = 2.24 nM) compared with donepezil (IC_50_ = 2.29 nM [4]) [34]. In addition, compound **1** can attenuate depressive symptoms accompanying Alzheimer’s disease by elevating serotonin levels. Regarding tacrine-bearing compounds, compound **17** (Figure 19) is the most promising due to its inhibitory activity (IC_50_ for *hu*AChE = 20.8 nM, IC_50_ for *hu*BChE = 0.0352 nM [48]), which is comparable to that of tacrine (IC_50_ for *hu*AChE is 3.16 nM and IC_50_ for *hu*BChE is 0.0267 nM [83]) [48]. In addition, compound **17** is not hepatotoxic, unlike tacrine, and possesses neuroprotective properties. By analyzing targets of MTDLs published between 2020 and 2024, it is clear that ChEs are the most popular. The second most popular target after ChEs are beta-amyloid aggregates and neurofibrillary tangles. An interesting representative of MTDL in this group is compound **49** (Figure 45), which indirectly contributes to the reduction of NFT formation by inhibiting tau kinases DYRK1A (IC_50_ = 111 nM) and GSK-3β (IC_50_ = 66 nM) [71] with high potency. Due to the importance of cholinergic neurotransmission in the impairment of memory and cognitive decline occurring in AD, future research for new anti-AD MTDLs will still focus on developing multifunctional ChE inhibitors. Additional targets, although less frequently considered, could include NMDARs, GSK-3β, and DYRK1A. It is also important to account for other conditions associated with AD, such as depression. Consequently, future MTDLs, besides influencing ChEs, could target SERT, 5-HTRs, or MAO-B. Regarding AD-related clinical trials, there are currently no MTDLs being tested. However, taking into account drugs that are currently undergoing or have been in clinical trials over the past four years, we can observe a shift in biological targets and types of drugs. Increasingly, so-called “small molecules” are being tested in clinical trials alongside monoclonal antibodies, which were previously more commonly associated with Alzheimer’s disease. An intriguing trend in today’s clinical trials is the focus on targeting neuroinflammation and mitigating cognitive decline. Nevertheless, the most frequently targeted entities in clinical trials remain beta-amyloid aggregates and tau protein [104,105,106,107,108,109,110,111,112,113,114,115,116,117,118,119,120,121,122,123,124,125]. Hence, we can hypothesize that the group of MTDLs with the greatest potential to reach clinical trials consists of compounds that possess anti-aggregative properties, improve cognitive functions, and provide neuroprotective effects. The latter two can be achieved, for instance, via cholinesterase inhibition, ROS scavenging, or the downregulation of interleukins, iNOS, and TNF-alpha. It is clear that novel compounds should aim to address the underlying causes of AD rather than merely alleviate the symptoms. In summary, there remains a significant need for new anti-Alzheimer agents, and finding them is a substantial challenge.

## Data Availability

Not applicable.
